# Fast-Response Calmodulin-Based Fluorescent Indicators Reveal Rapid Intracellular Calcium Dynamics

**DOI:** 10.1038/srep15978

**Published:** 2015-11-03

**Authors:** Nordine Helassa, Xiao-hua Zhang, Ianina Conte, John Scaringi, Elric Esposito, Jonathan Bradley, Thomas Carter, David Ogden, Martin Morad, Katalin Török

**Affiliations:** 1 Institute of Cardiovascular and Cell Science, St George’s, University of London, London SW17 0RE, UK; 2 Department of Regenerative Medicine and Cell Biology, Medical University of South Carolina, Charleston SC 29425, USA; 3 MRC National Institute for Medical Research, London NW7 3RJ, UK; 4 Laboratoire de Physiologie Cérébrale, Centre National de la Recherche Scientifique and Université Paris Descartes, 75006 Paris, France

## Abstract

Faithful reporting of temporal patterns of intracellular Ca^2+^ dynamics requires the working range of indicators to match the signals. Current genetically encoded calmodulin-based fluorescent indicators are likely to distort fast Ca^2+^ signals by apparent saturation and integration due to their limiting fluorescence rise and decay kinetics. A series of probes was engineered with a range of Ca^2+^ affinities and accelerated kinetics by weakening the Ca^2+^-calmodulin-peptide interactions. At 37 °C, the GCaMP3-derived probe termed GCaMP3_*fast*_ is 40-fold faster than GCaMP3 with Ca^2+^ decay and rise times, *t*_1/2_, of 3.3 ms and 0.9 ms, respectively, making it the fastest to-date. GCaMP3_*fast*_ revealed discreet transients with significantly faster Ca^2+^ dynamics in neonatal cardiac myocytes than GCaMP6f. With 5-fold increased two-photon fluorescence cross-section for Ca^2+^ at 940 nm, GCaMP3_*fast*_ is suitable for deep tissue studies. The green fluorescent protein serves as a reporter providing important novel insights into the kinetic mechanism of target recognition by calmodulin. Our strategy to match the probe to the signal by tuning the affinity and hence the Ca^2+^ kinetics of the indicator is applicable to the emerging new generations of calmodulin-based probes.

Visualising Ca^2+^ fluxes in cells, tissues or free-moving animals offers an important way of gaining understanding of a wide range of physiological mechanisms including cardiac function, cell signalling and neuronal network activity. Monitoring fast or high frequency Ca^2+^ transients, characteristic of excitable cells, requires rapid kinetics with millisecond time resolution. Genetically encoded fluorescent Ca^2+^ indicators (GECI) are being developed to fulfil these roles and have become important tools for activity studies using targeted expression in cultured cells and tissues and in live transgenic animals^1^.

Green fluorescent protein - calmodulin protein - (GCaMP)-type GECI are based on a circularly permutated EGFP molecule (cpEGFP)^2^ flanked at the N and C termini by the smooth muscle myosin light chain kinase derived calmodulin binding peptide (RS20) and calmodulin (CaM), respectively^3^. The preferred protonated state of cpEGFP has low brightness. Its intrinsic fluorescence is enhanced by Ca^2+^ binding, which causes the Ca^2+^.CaM.RS20 complex to form, stabilizing the highly fluorescent, deprotonated form of GCaMP by blocking solvent access to the chromophore^4^,^5^.

Several generations of GCaMP-type GECI obtained by mutagenesis have presented improvements in brightness, signal-to-noise ratio and photostability^6^,^7^,^8^,^9^,^10^,^11^,^12^,^13^,^14^,^15^,^16^. Mutations to the linker regions and to the cpEGFP domain itself resulted in greater fluorescence enhancements on Ca^2+^ binding by shifting the equilibrium towards the deprotonated state and/or increasing the brightness of the deprotonated form. These mutations, however, left unchanged the high affinity (equilibrium dissociation constant *K*_d_ in the range of hundreds of nM) and concomitant slow response kinetics of the probes to Ca^2+^. There has been a lack of insight into the onset kinetics and slow fluorescence decay rates of 1–5 s^−1^ have constrained tracking rapid processes. In excitable cells monitoring fast Ca^2+^ fluxes requires millisecond time resolution and thus fluorescence decay and rise rates of up to 1000 s^−1^. These issues have begun to be addressed^17^,^18^, however, to-date there are no GECI probes sufficiently rapid for accurate tracking of fast or high-frequency Ca^2+^ transients associated with synaptic transmission and activation of skeletal and cardiac muscle.

Our aim was to generate fast derivatives of GCaMP3 by rational design with Ca^2+^ response kinetics on the millisecond time scale and to understand the kinetic mechanisms by which GCaMPs respond to Ca^2+^ concentration changes. Using both excitable and non-excitable cellular model systems we compared the response kinetics of the newly developed probes with those of the chemical dye Fura-2, GCaMP3 and GCaMP6f. Imaging with the newly developed probe GCaMP3_*fast*_ revealed significantly faster Ca^2+^ response kinetics than previously seen with GECI.

## Results

### Design of fast-response GCaMPs

The high affinity of GCaMPs for Ca^2+^ (*K*_d_ of 100 - 300 nM^8^,^11^,^15^) is determined by the equilibrium of the Ca^2+^.CaM.RS20 peptide complex. We have previously shown that the mutation of the Trp residue to Tyr in a RS20-related peptide increases the *K*_d_ of the complex for Ca^2+^.CaM^19^,^20^. Moreover, disabling individual EF-hand Ca^2+^ binding sites of CaM by single point mutations (Asp → Ala) in the first loop residue of each EF-hand increased the *K*_d_ of CaM for Ca^2+^ 21,22. As a consequence, Ca^2+^ dissociation from complexes of Ca^2+^-bound EF-hand mutated CaM with a peptide derived from αCaMKII (CaMKIIP) was significantly accelerated. Based on these observations our hypothesis was that fast Ca^2+^ response rates can be achieved for GCaMPs by weakening the Ca^2+^.CaM.RS20 interaction by the peptide and EF-hand mutations described above.

**Figure 1:**
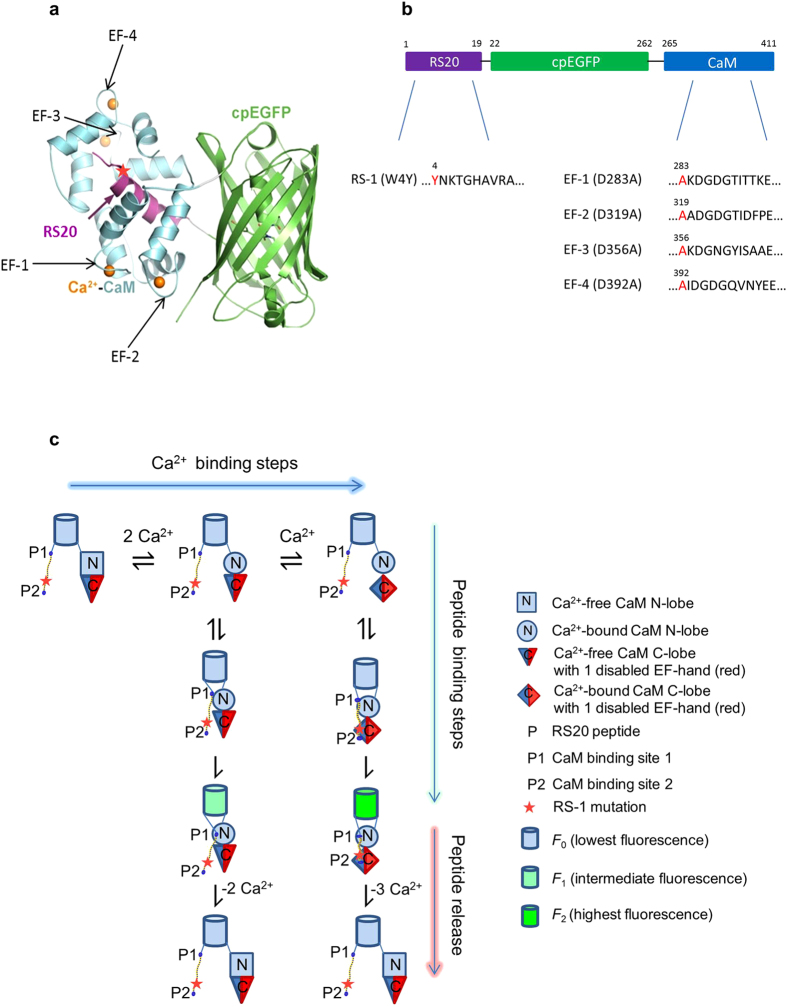
Design of mGCaMPs and kinetic mechanism of Ca^2+^-induced fluorescence changes. (**a**) Crystal structure of monomeric GCaMP2 in a Ca^2+^-bound form with the RS20 peptide (purple), cpEGFP (green) and CaM (blue) (adapted from Akerboom *et al.*^4^). The approximate position of the mutations in the CaM EF-hand Ca^2+^-binding sites is shown by arrows and in the RS20 peptide by a red star. (**b**) Domain structure of GCaMP3. The mutated residues and their positions in the primary structure are highlighted in red. (**c**) Schematic model of the kinetic mechanism of Ca^2+^-induced fluorescence of GCaMP3_*fast*_. For the GCaMP3_*fast*_ mutant the disabled CaM C-lobe EF-hand is painted red and the RS-1 mutation is indicated by a red star. For fitting the model to the data, Ca^2+^ binding to EF-3, corresponding to equation (5) of Supplementary Fig. S14, was omitted. Ca^2+^ binding to the CaM N-lobe is followed by the development of the first fluorescent state (*F*_1_) by peptide binding at the P1 site and a structural change. N-lobe Ca^2+^-bound CaM proceeds to full Ca^2+^ binding and complete peptide binding at the P2 site, which, following a structural rearrangement, presents the second fluorescent state (*F*_2_). Ca^2+^ dissociation occurs directly from the two fluorescent states. Fitted parameters are shown in Supplementary Table S3. For GCaMP3_*bright*_, the diagram without the red star applies and equation (6) was absent for the data fitting.

Our strategy therefore was to disable from one to three of the four EF-hand Ca^2+^ binding sites in the CaM sequence in GCaMP3-derived proteins, and to investigate the effect of Trp → Tyr mutation in the RS20 target peptide sequence, alone, and in combination with the EF-hand mutations (Fig. 1a,b).

Triple EF-hand mutations in all cases resulted in a loss of fluorescence response to Ca^2+^ binding, and were not studied further. The following single and double EF-hand mutants were generated and characterised – the numbers identify the mutated EF-hand(s): EF-1, EF-2, EF-3, EF-4, EF-1:2, EF-1:3, EF-1:4, EF-2:3, EF-2:4, EF-3:4. The Trp → Tyr peptide mutant was termed RS-1 and the peptide mutation combined with mutation of the first EF-hand gave RS-1 EF-1 and so on, resulting all together in 21 GCaMP3 mutants (mGCaMP3). The same EF-hand mutagenesis strategy that was applied to GCaMP3 was also tested on two of its derivatives developed by Zhao *et al.*^11^, giving 20 additional mutants. The properties of 41 mutants are presented in Supplementary Materials, Supplementary Fig. S3, S5–S8, S11 and Supplementary Tables S1–S4).

Detailed characterisation is presented below of two mGCaMP3 probes: GCaMP3_*fast*_ (mGCaMP3 RS-1 EF-3) with the fastest Ca^2+^ response times is promising for monitoring high-frequency Ca^2+^ transients and GCaMP3_*bright*_ (mGCaMP3 EF-4) with the highest fluorescence dynamic range. Other mutants presented in Supplementary Table S2 show features such as high *K*_d_ (mM range) that are potentially useful for monitoring Ca^2+^ in organelles. Indicator responses occurred on the millisecond time scale and thus Ca^2+^ response kinetics were assessed by stopped-flow. A kinetic model proposed to explain the Ca^2+^ response mechanism of GCaMP3 and mGCaMP3 is illustrated on GCaMP3_*fast*_ and GCaMP3_*bright*_ (without the red star at P2 that signifies the RS-1 mutation) (Fig. 1c and Supplementary Fig. S13). Fitted parameters for GCaMP3_*fast*_ and GCaMP3_*bright*_ and further five mGCaMP3 probes of potential interest in physiological studies are presented (Supplementary Table S3).

### Biophysical characterisation of mutant GCaMP3 probes

Mutations in the peptide and CaM EF-hands did not affect the stability of the proteins. Fluorescence emission of GCaMP3_*fast*_ was stable at 37 °C for four hours, in the absence and presence of Ca^2+^ (Supplementary Fig. S1). Relative fluorescence of GCaMP3 in the presence of Ca^2+^ 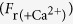 was 6.3 compared to Ca^2+^-free GCaMP3, the fluorescence intensity of which was defined to have relative fluorescence 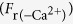 of 1. The GCaMP3_*fast*_ and GCaMP3_*bright*_ probes had somewhat lower 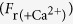 and 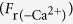 values than GCaMP3 but their Ca^2+^ binding-induced fluorescence enhancement was greater: 9-fold for GCaMP3_*fast*_ and 14-fold for GCaMP3_*bright*_ (Table 1). Quantum yield (Φ) measurements revealed that the mutations did not cause large changes compared to GCaMP3. Using the value Φ_+Ca_^2+^ of 0.44 as a reference we obtained Φ_−Ca_^2+^ of 0.21, essentially the same as previously described^11^. GCaMP3_*fast*_ had Φ_+Ca_^2+^ of 0.42 and Φ_−Ca_^2+^ of 0.14, while Φ_+Ca_^2+^ was 0.35 and Φ_−Ca_^2+^ 0.09 for GCaMP3_*bright*_. p*K*_a_ values were 7.5–8 in the presence and ~8.2 in the absence of Ca^2+^ for all three probes (Supplementary Fig. S2).

Simultaneous mutation of both C-lobe Ca^2+^ binding sites (mGCaMP3 EF-3:4) moderately reduced the fluorescence dynamic range (Supplementary Table S1). However, mutation of either of the N-terminal Ca^2+^ binding sites EF-1 or EF-2 was severely detrimental to the Ca^2+^ response and double mutant EF-1:2 was essentially non-responsive to Ca^2+^ (Supplementary Table S2). The N-lobe Ca^2+^ binding sites are thus critical for functional probes, whereas mutation of C-lobe sites can improve the fluorescence dynamic range of GCaMPs.

**Figure 2:**
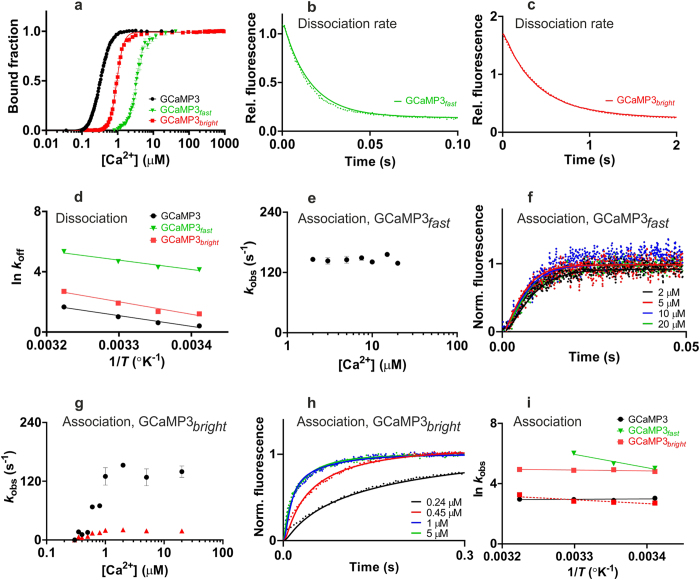
Biophysical characterisation of GCaMP3_*fast*_ and GCaMP3_*bright*_. (**a**) Equilibrium Ca^2+^ titrations for: GCaMP3 (●), GCaMP3_*fast*_ (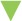) and GCaMP3_*bright*_ (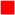). Fluorescence changes are normalised to *F*_0_ of 0 and *F*_max_ of 1 and fitted to the Hill equation. Fitted curves are represented by solid lines overlaying the data; (**b**,**c**) Dissociation kinetics of GCaMP3_*fast*_(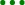) and GCaMP3_*bright*_ (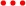) fitted to single exponentials. Intensities are relative to Ca^2+^-bound GCaMP3. Experimental data (dotted lines) are overlaid with fitted curves (solid lines) generated using the parameters shown in Supplementary Table S3 fitted to the model in Supplementary Fig. S13; (**d**) Arrhenius plots of the observed dissociation rates of GCaMP3 (●), GCaMP3_*fast*_ (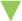) and GCaMP3_*bright*_ (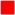) recorded at 20, 25, 30, 37 °C; (**e–h**) Association kinetics. (**e**) Plot of the [Ca^2+^] dependence of the observed rate(s) (*k*_obs_) for GCaMP3_*fast*_; (**f**) stopped-flow records for GCaMP3_*fast*_ at 2 μM (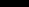), 5 μM (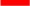), 10 μM (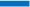) and 20 μM (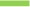) [Ca^2+^]. Fluorescence changes are normalised to *F*_0_ of 0 and *F*_max_ of 1. Experimental data (dotted lines) are overlaid with fitted curves (solid lines) generated using the parameters shown in Supplementary Table S3 fitted to the model in Supplementary Fig. S13; (**g**) Plot of the [Ca^2+^] dependence of the observed rate(s) (*k*_obs_) for GCaMP3_*bright*_; (**h**) stopped-flow records for GCaMP3_*bright*_ at 0.24 μM (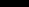), 0.45 μM (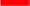), 1 μM (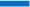) and 5 μM (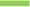) [Ca^2+^]. Experimental data (dotted lines) are overlaid with fitted curves (solid lines) generated using the parameters shown in Supplementary Table S3 fitted to the model in Supplementary Fig. S13. As GCaMP3_*bright*_ has biphasic kinetics, the slower rate is shown in red and the faster rate in black; (**i**) Arrhenius plots of the observed association rates of GCaMP3 (●), GCaMP3_*fast*_ (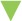) and GCaMP3_*bright*_ (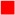) recorded at 20, 25, 30, 37 °C. For mGCaMP3 with biphasic kinetics, the slower process is shown in dotted line and the faster process is in solid line.

Most of the mGECI generated had higher *K*_d_ values and faster fluorescence decay rates than the equivalent wild type protein (Fig. 2a–c, Supplementary Fig. S3–S5 and Supplementary Tables S1, S2). Equilibrium dissociation constants (*K*_d_) obtained by Ca^2+^ titration were increased from 330 nM for GCaMP3 to 1–3 μM for GCaMP3_*fast*_ and GCaMP3_*bright*_. Cooperativity of Ca^2+^ binding, characterised by a Hill coefficient (*n*) of 3.2 for GCaMP3 was higher, 4.3 and 4.7, for GCaMP3_*fast*_ and GCaMP3_*bright*_, making their switch-like Ca^2+^ response steeper than that of GCaMP3 (Fig. 2a and Table 1).

The kinetics of Ca^2+^ response of GCaMP3 and mGCaMP3 were measurable by stopped-flow kinetics with the limitation of a ~1.5 ms response time allowing the measurement of rates up to ~500 s^−1^. The most significant improvements in the Ca^2+^ dissociation kinetics of GCaMP3 were achieved by the combination of the RS-1 and EF-3 mutations. At 20 °C, the *t*_1/2_ of 461 ms for Ca^2+^ dissociation of GCaMP3 was reduced to 11 ms (3.3 ms at 37 °C) with the GCaMP3_*fast*_ probe (*t*_1/2_ values were calculated from the dissociation rates measured at the indicated temperatures, Fig. 2b,d, Table 1 and Supplementary Fig. S4).

Investigation of the kinetics of fluorescence changes upon Ca^2+^ association revealed novel features. First, a limiting rate (*k*_on(lim)_) was observed for all GECI and mGECI generated in this study. For GCaMP3 at physiological ionic strength and 20 °C the rate saturated at 20 s^−1^ (Supplementary Fig. S4 and Table 1), corresponding to a rise time (*t*_1/2_) of 35 ms. The GCaMP3_*fast*_ and GCaMP3_*bright*_ mutations increased the limiting rate to ~140 s^−1^ (20 °C). Second, the observed rates showed a steep dependence on [Ca^2+^]. Consistent with their increased cooperativity for Ca^2+^ compared to GCaMP3, the observed rates of the two mutants switched to a limiting maximum over a narrow range of [Ca^2+^] around their dissociation constants (Fig. 2e,g and Table 1). A number of other mGECI also showed high cooperativity (Supplementary Fig. S6–S8 and Supplementary Tables S1, S2). Third, biphasic fluorescence rise kinetics were found in GCaMP3_*bright*_ with saturating rates at 135 s^−1^ and 19 s^−1^. Each phase represented 50% of the total amplitude, revealing the existence of two distinct fluorescent conformers (Figs 1c and 2g and Table 1). Fourth, strikingly, the limiting fluorescence rise rates of GCaMP3 and GCaMP3_*bright*_ were essentially independent of temperature in the temperature range of 20 °C to 37 °C. The faster and slower phases of GCaMP3_*bright*_ saturating at 135 s^−1^ and 19 s^−1^ at 20 °C increased only to 139 s^−1^ and 26 s^−1^ at 37 °C. In contrast, GCaMP3_*fast*_ showed marked temperature dependences with rates increased to 411 s^−1^ at 30 °C. The time resolution did not permit direct measurement at 37 °C. The limiting rate was extrapolated from Arrhenius plots giving a fluorescence rise rate of 743 s^−1^ for GCaMP3_*fast*_ at 37 °C (Fig. 2i and Table 1). GCaMP3_*fast*_ and GCaMP3_*bright*_ presenting the fastest responses to Ca^2+^ in combination with the highest dynamic ranges were selected for further physiological characterization.

### Monitoring fast Ca^2+^ transients in neonatal cardiac myocytes with GCaMP3_*fast*_

**Figure 3:**
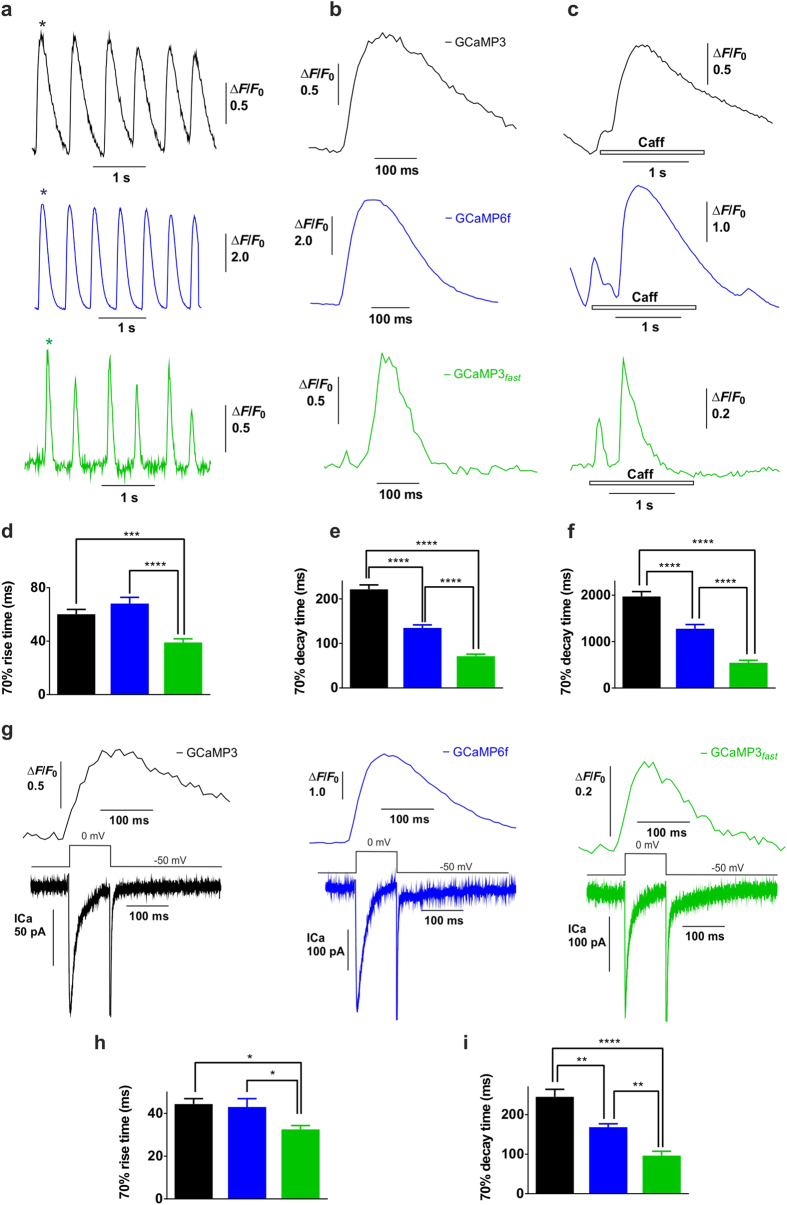
Comparison of the kinetics of GCaMP3, GCaMP6f and GCaMP3_*fast*_ in neonatal cardiomyocytes. Recorded by GCaMP3 (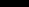   ), GCaMP6f (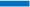) and GCaMP3_*fast*_ (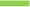) are: (**a**) spontaneous whole-cell cytosolic Ca^2+^ signals in Ad-CMV-virus infected cells imaged confocally at 120 Hz (n ≥ 10); (**b**) expanded beats, marked by stars; (**c**) representative traces of Ca^2+^ release after application of 3 mM caffeine (n ≥ 9), imaged confocally at 30 Hz; (**d**) average values of 70% Ca^2+^ rise time in spontaneously beating cardiac myocytes; (**e**) average values of 70% Ca^2+^ decay time in spontaneously beating cardiac myocytes; (**f**) average values of 70% relaxation time after caffeine application; (**g**) Ca^2+^ current activated Ca^2+^ release (CICR) in voltage clamped neonatal cardiomyocytes (n ≥ 7), imaged confocally at 120 Hz. Cells are voltage clamped at −50 mV and depolarized to 0 mV; (**h**) average values of depolarization-activated 70% Ca^2+^ rise time; (**i**) average values of depolarization-activated 70% relaxation time.

Neonatal cardiac myocytes were chosen for the fast kinetics and physiological relevance of their Ca^2+^ transients. In spontaneously beating neonatal cardiac myocytes, where spontaneous pacing is seen as rhythmic Ca^2+^ releases, comparison of the fluorescence rise times of GCaMP3 (60 ms), GCaMP6f (68 ms) and GCaMP3_*fast*_ (39 ms) showed that GCaMP3_*fast*_ was ~1.5-fold faster than either of the other two probes (Fig. 3a,b,d). The fluorescence decay times for GCaMP3 (222 ms), GCaMP6f (135 ms) and GCaMP3_*fast*_ (71 ms) were even more markedly improved, GCaMP3_*fast*_ was 3-fold faster than GCaMP3 and twice as fast as GCaMP6f (Fig. 3a,b,e). Response kinetics were similarly strikingly improved to caffeine stimulation with fluorescence decay times 4-fold reduced for GCaMP3_*fast*_ compared to GCaMP3 and 2-fold reduced compared to GCaMP6f (Fig. 3c,f). Remarkably, significantly faster kinetics of intracellular Ca^2+^ rise and fall were recorded in patch-clamped myocytes on activation of I_Ca_-gated Ca^2+^ release (CICR) with GCaMP3_*fast*_ compared to the other two probes (rise-times 44 ms for GCaMP3 and 43 ms for GCaMP6f compared to 33 ms for GCaMP3_*fast*_ and decay-times of 245 ms for GCaMP3 and 168 ms for GCaMP6f compared to 95 ms for GCaMP3_*fast*_) (Fig. 3g–i). While Δ*F*/*F*_0_ is a modest 0.2 when measured throughout an entire beating cell (Fig. 3g), the fluorescence dynamic range of localized perinuclear Ca^2+^ signals recorded at 120 Hz, at the hotspots reaches ~1 (Supplementary Fig. S9).

### Live cell imaging of GCaMP3_*fast*_ and GCaMP3_*bright*_ in human endothelial cells and embryonic kidney cells

**Figure 4:**
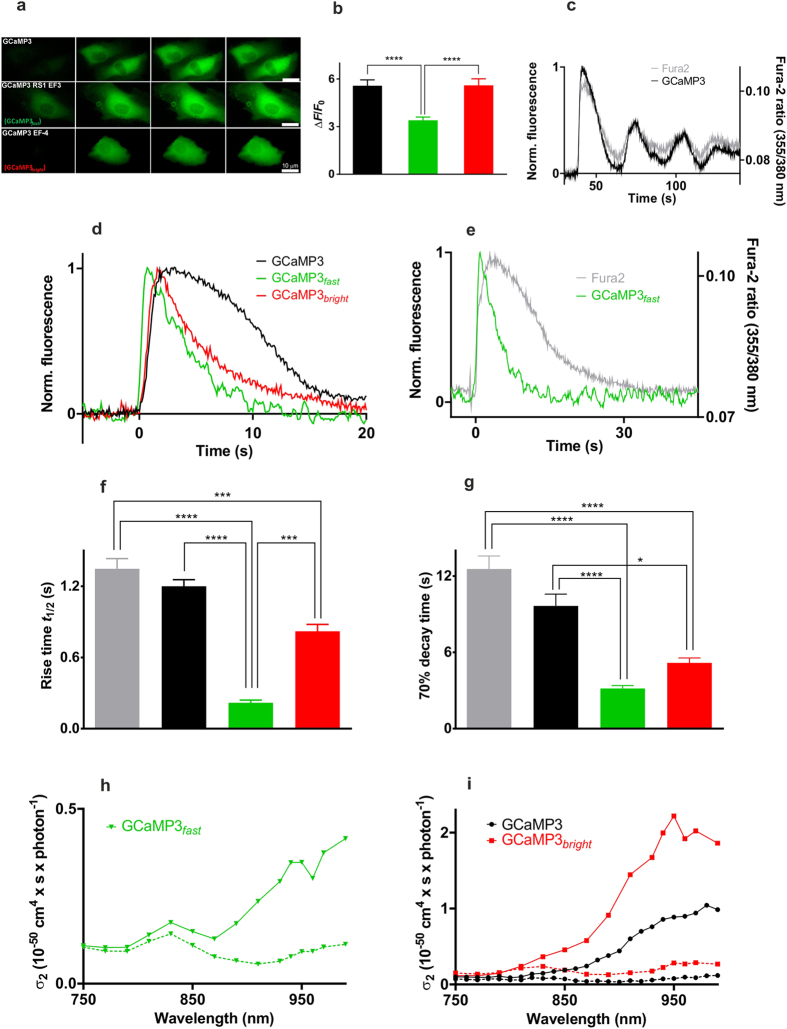
Characterisation and application of selected mGCaMP3 in HUVEC. Fura-2 (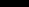), GCaMP3 (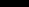), GCaMP3_*fast*_ (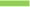) and GCaMP3_*bright*_ (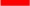): (**a**) signals are recorded by live cell imaging. Ca^2+^ influx is evoked by ionomycin. Image frames taken at times 0.4 s, 0.8 s, 2.67 s and 4.40 s are shown. (**b**) fluorescence dynamic ranges following ionomycin treatment (n ≥ 12); (**c–e**) representative records following exposure to 2 μM ATP. Comparison of: (**c**) GCaMP3 with Fura-2; (**d**) GCaMP3 with GCaMP3*_fast_* and GCaMP3*_bright_*; (**e**) GCaMP3*_fast_* with Fura-2. Fluorescence rise times (**f**) and decay times (**g**) for Fura-2, GCaMP3, GCaMP3_*fast*_ and GCaMP3_*bright*_ (n ≥ 5) following ATP stimulation. Two-photon excitation spectra of (**h**) GCaMP3_*fast*_ (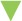); (**i**) GCaMP3 (●) and GCaMP3_*bright*_ (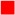). Spectra are taken in the presence (solid lines) and in the absence (dotted lines) of Ca^2+^.

Imaging the fluorescence of GCaMP3_*fast*_ and GCaMP3_*bright*_ in the cellular environment of human umbilical vein endothelial cells (HUVEC) and HEK293T cells revealed no detectable damage to cell viability. Fluorescence dynamic ranges (Δ*F*/*F*_0_) of mGCaMP3 expressed in the cytosol of HUVEC during acute exposure to ionomycin remained high with values of 3.4 and 5.6 for GCaMP3_*fast*_ and GCaMP3_*bright*_ respectively, comparable to GCaMP3 (Fig. 4a,b and Supplementary Tables S1, S2).

The kinetics of GCaMP3 and mGCaMP3 fluorescence changes were compared with those of the Ca^2+^ dye Fura-2 by simultaneous imaging. Extracellular ATP-induced Ca^2+^ transients were reported with similar kinetics by both Fura-2 and GCaMP3 in HUVEC and HEK293T cells (Fig. 4c and Supplementary Fig. S10). The fluorescence of both Fura-2 and GCaMP3 reached a plateau before slowly declining with complex kinetics (Fig. 4c–e and Supplementary Fig. S10). In contrast, GCaMP3_*fast*_ and GCaMP3_*bright*_ fluorescence response to hormone stimulation was characterised by rise to a sharp peak followed by exponential decline to baseline (Fig. 4d,e and Supplementary Fig. S10). The fluorescence rise times for GCaMP3_*fast*_ and GCaMP3_*bright*_ and the decay time for GCaMP3_*fast*_ were significantly faster than either Fura-2 or GCaMP3 (Fig. 4f,g and Supplementary Fig. S10). GCaMP3_*fast*_ responded with a 0.22 ± 0.08 s rise and a 3.2 ± 0.4 s decay time in ATP stimulated HUVEC (Fig. 4f,g). Δ*F*/*F*_0_ values are reciprocal to *K*_d_-s indicating Fura-2 and GCaMP3 to be saturated. In contrast, the lower affinity GCaMP3_*fast*_ and GCaMP3_*bright*_probes with less than maximum fluorescence enhancement show partial binding. These data altogether suggest comparable peak values for [Ca^2+^] in the range 0.5–1 μM (Supplementary Fig. S10).

### Two-photon cross-sections of mGCaMPs of mGECI

Because animal tissue is transparent to infrared light, two-photon excitation microscopy has become a crucial tool for tissue and *in vivo* fluorescence imaging. Two-photon excitation fluorescence cross-section (σ_2_) or two-photon absorption represents the absorption coefficient for the simultaneous absorption of two photons for the excitation of a fluorophore. Although the depth of penetration is limited by the wavelength, the signal strength and signal-to-noise ratio will depend on the absolute value of σ_2_. Hence we determined the σ_2_ values the novel mGECI in the presence and absence of Ca^2+^ relative to fluorescein^23^ (Fig. 4h,i and Supplementary Fig. S11). Compared to the 21 × 10^−50^ cm^4^.s.photon^−1^ value for fluorescein, GCaMP3 had the lowest σ_2_ value in the absence of Ca^2+^ (0.06 × 10^−50^ cm^4^.s.photon^−1^). In the presence of Ca^2+^ the σ_2_ value increased to 0.76 × 10^−50^ cm^4^.s.photon^−1^. The GCaMP3_*fast*_ probe had a ~5-fold increase upon Ca^2+^ binding. The brightest probe with the greatest fluorescence dynamic range under two-photon excitation was GCaMP3_*bright*_ with a ~10-fold increase and higher σ_2_ values, 0.22 × 10^−50^ cm^4^.s.photon^−1^ and 2.0 × 10^−50^ cm^4^.s.photon^−1^, in the presence and absence of Ca^2+^, respectively. It is envisaged that the probes developed here will be useful to study fast signalling processes, such as the Ca^2+^ changes associated with neuronal activity in tissues and *in vivo.*

## Discussion

Based on previous studies^19^,^21^ we devised a rational approach to alter the kinetic properties of GCaMPs for better detection of fast Ca^2+^ signalling events. Disabling the function of individual Ca^2+^ binding sites by single point mutations in the CaM portion of GCaMPs was expected to lower the Ca^2+^ affinity and accelerate Ca^2+^ kinetics^21^. Weakening the Ca^2+^.CaM - peptide interaction by mutation of the RS20 peptide was expected to further lower Ca^2+^ affinity as substitution of the Trp residue with Tyr was known to increase the *K*_d_ for peptide binding to Ca^2+^.CaM^19^,^20^. Consistent with our predictions, CaM EF-hand and peptide RS-1 mutations of GCaMP3 resulted in increasing the *K*_d_ for Ca^2+^ binding from 330 nM to 1–3 μM. Fura-2 and GCaMP3 with sub-μM *K*_d_ for Ca^2+^ showed slow and complex Ca^2+^ response kinetics in ATP-stimulated in HUVEC and HEK293T cells, indicating probe saturation. GCaMP3_*fast*_ and GCaMP3_*bright*_ revealed that the hormone-induced Ca^2+^ transients were faster than when reported either by Fura-2 or GCaMP3. Our data thus show that the amplitude and kinetics of hormone-evoked intracellular Ca^2+^ transients are better matched by Ca^2+^ probes with a μM working range, like GCaMP3_*fast*_and GCaMP3_*bright*_, than by those with *K*_d_ values for Ca^2+^ of 200–300 nM.

A major limitation of GCaMP3 in faithfully tracking Ca^2+^dynamics has been its slow Ca^2+^ dissociation (*t*_1/2_ of 131 ms) at 37 °C. Recently reported probes GCaMP6 ‘fast’ (GCaMP6f)^15^ (*t*_1/2_ of 66 ms (37 °C) (N. Helassa and K. Török, unpublished data)), the RS09 GCaMP3 mutant^18^ (*t*_1/2_ of 25 ms, (37 °C)) and fast-GCaMP6f-RS09 (*t*_1/2_ of 20 ms (37 °C))^24^ have faster dissociation kinetics. Our GCaMP3_*fast*_ probe with a *t*_1/2_ of 3.3 ms for fluorescence decay at 37 °C is the fastest currently available GECI.

The maximal fluorescence onset rates determine the time resolution at which fluorescence changes are detected by the probes. Response times of >50 ms for GCaMP6f, fast-GCaMP6f-RS06 and fast-GCaMP6f-RS09 were measured in mammalian brain slices. When compared to times to peak of Ca^2+^ transient measurements with the fast linear-response fluorescent dye Furaptra of 36 ms in patch-clamped cardiac myocytes^25^, 6.3 ms in electrically stimulated frog skeletal muscle^26^, 13 ms in endothelial cells for IP_3_-evoked Ca^2+^ release^27^ and 35 ms in Purkinje neurons for IP_3_-evoked Ca^2+^ release^28^, it is evident that the kinetics of GCaMP3_*fast*_are not limiting the detection of fast Ca^2+^ transients in cells. The RS-1 and EF-hand mutations resulted in up to 40-fold higher rates compared to GCaMP3, taking the response times of GCaMP3_*fast*_ down to just under 1 ms at 37 °C.

We compared the response kinetics of GCaMP3_*fast*_ to GCaMP3 and GCaMP6f in a fast response physiological system. In neonatal cardiac myocytes Ca^2+^ rise and decays times were significantly faster during spontaneous beating, after caffeine stimulation and in I_Ca_-gated Ca^2+^ release when recorded using GCaMP3_*fast*_. In contrast to GCaMP3 and GCaMP6f, GCaMP3_*fast*_ revealed entirely discrete Ca^2+^ oscillations in spontaneously beating cardiac myocytes with a 2.5-fold shorter duration (~210 ms) compared to GCaMP6f (~535 ms) for each oscillatory calcium spike. GCaMP3_*fast*_ thus shows greater time resolution and is more suitable for tracking fast Ca^2+^ transients than GCaMP6f. In cardiac myocytes following 50 ms depolarisation, Furaptra had 29 ms time to peak rise and 66 ms decay times^25^. Our measured rise and decay times of GCaMP3_*fast*_ in cardiac myocytes following 100 ms depolarisation were a comparable 33 ms and 96 ms, respectively (Fig. 3h,i), suggesting that the Ca^2+^ rise and fall times measured in cardiac myocytes were not limited by the Ca^2+^ response kinetics of GCaMP3_*fast*_.

Extensive quantitative studies on [Ca^2+^] in cultured endothelial cells have been previously made with the low affinity indicator Furaptra^27^,^29^. The rising phase of Ca^2+^ transients in endothelial cells is fast and the peak [Ca^2+^] high, reaching 20 μM, as shown in experiments either with ATP stimulation or intracellular store release by photo-released InsP_3_^27^ or for acetylcholine^29^. The range of [Ca^2+^] rise rates had mean 5.3 μM/s in 100 μM ATP with range from cell to cell in 8 cells of 1–16 μM/s. The rise times indicated by the low affinity Furaptra were 1.9 ± 1.5 s. Similar large amplitude (6–35 μM) fast (time to peak 0.8–2.7 s) transients were seen with acetylcholine with decay times of 1.6–9.1 s. GCaMP3_*fast*_ responded with a faster 0.22 ± 0.08 s rise and a comparable 3.2 ± 0.4 s decay time in ATP stimulated HUVEC (Fig. 4d-g).

The basic considerations that go into choosing a Ca^2+^-indicator dye for a specific purpose include brightness, dynamic range, calibration, molecular targeting and resistance to bleaching. The flexibility in the design of genetically engineered probes provides the experimentalist with the option to choose a probe that meets specific needs, where speed and high Hill coefficient may be exactly what is needed in some situations, while brightness, linearity and sensitivity to small signals are the pre-requisites for addressing variations in baseline [Ca^2+^]. It is evident that the affinity and response kinetics of the probes are critical to optimum reporting. Firstly, the synthetic fluorescent Ca^2+^ probes have diffusion limited on kinetics in the range of ~5 × 10^8^ M^−1^ s^−1^, and the commonly used Fluo-3 (*k*_on_ = 7 × 10^8^ M^−1^ s^−1^, *k*_off_ = 370 s^−1^, *K*_d_ = 370 nM, *n* = 1)^30^ and Fura-2 (*k*_on_ = 4 × 10^8^ M^−1^ s^−1^, *k*_off_ = 100 s^−1^, *K*_d_ = 260 nM, *n* = 1)^30^ dyes. GCaMP-type GECI probes have more complex Ca^2+^ response kinetics in that they have a limiting rate of onset. Faster response kinetics by GCaMP3_*fast*_ and GCaMP3_*bright*_ were achieved by an increase in both the limiting rate of onset and the rate of decay. Secondly, having low *K*_d_-s may often result in saturation, e.g. when the Ca^2+^ stores are fully released. Fast kinetics are achieved by increasing the *K*_d_ and consequently also the off-rate. However, when the *K*_d_ does not match the signal, e.g. *K*_d_ ≫ [Ca^2+^] peak, then the fluorescence dynamic range is reduced. Thirdly, the genetically engineered dyes can be targeted to specific proteins and therefore be used to probe micro-domains of Ca^2+^ that are of relevance for privileged communications between subcellular organelles. In this case speed and low affinity are both required (e.g. CalciumGreen-5N: *k*_on_ = 2 × 10^8^ M^−1^ s^−1^, *k*_off_ = 9,000 s^−1^, *K*_d_ = 23 μM, *n* = 1)^30^, since the local Ca^2+^ concentration may increase to tens or hundreds of μM, but do so only briefly before being dissipated by diffusion.

Physiological [Ca^2+^] concentration ranges roughly fall into three groups: 100 nM to 1 μM, 1 μM to 20 μM (cytosolic) and 100 μM to mM (subcellular organelles and extracellular matrix). Useful indicators (example probes in Supplementary Table S1) either have a *K*_d_ that is slightly higher than the range of interest (with a moderate dynamic range) or have a high enough *K*_d_ ([Ca^2+^] ~ × 10) (example probes in Supplementary Table S2) for linear response and suitable for quantification provided their fluorescence dynamic range is high enough to give a good signal at fractional saturation. GCaMP-type indicators most of which have a switch-like response indicate signals best at concentrations which are around their *K*_d_. This is illustrated well in cardiac myocytes where GCaMP3_*fast*_ and GCaMP3_*bright*_ (*K*_d_-s of 1–3 μM) indicate 0.5–1 μM [Ca^2+^] with the greatest fidelity when compared with the high affinity GCaMP3, GCaMP6f and Fura-2 which distort the time course of Ca^2+^ signals due to saturation and slow equilibration (Figs 3 and 4 and Supplementary Fig. S9). Although GCaMP6f and Fura-2 have fast enough Ca^2+^ on rates, the onset of the signal is similarly affected as for GCaMP3 which has a limiting response rate of 20 s^−1^. Faster off rates avoid prolonging buffering and studies with low affinity indicators reveal that even large Ca^2+^ transients are cleared at a high rate^27^,^29^. The benefit of faster off rates is clearly seen in the detection of local Ca^2+^ hotspots in cardiac myocytes on the 8–20 ms time scale (Supplementary Fig. S9).

Our kinetic characterisation revealed that the fluorescence response of GCaMPs to Ca^2+^ elevation is close to all-or-none at a limiting rate. The switch occurs around the *K*_d_ for Ca^2+^. With this type of indicator, Ca^2+^ transients are counted rather than measured. The affinity-lowering mutations increased the limiting on rate as well as decay rate bringing the on rate into the low millisecond range.

We now discuss our findings regarding the kinetic mechanism of calmodulin-based GECI. Since the fluorescence of EGFP in effect reports the interaction of Ca^2+^.CaM with the RS20 peptide, it became possible to gain novel insights into the mechanism by which Ca^2+^.CaM recognizes and engages with its targets. First, the established cooperativity of Ca^2+^ binding is shown to be based on highly cooperative kinetics of Ca^2+^ association. Second, fluorescence onset occurs with a limiting maximum rate. Third, evidence for a partial CaM N-lobe only-attached peptide complex is shown by mGCaMP3 EF-3:4.

Apparently greater degrees of cooperativity beyond that predicted by the number of Ca^2+^ sites alone suggests that conformational changes associated with the peptide binding processes strengthen Ca^2+^ binding by the Ca^2+^.CaM.cpEGFP.RS20 mutant complexes. Dimerization could be a contributing factor. However it seems less likely as the mutations are not in the regions of the molecule, i.e. the cpEGFP domain, which are responsible for dimerization. Strikingly, saturating rates at high [Ca^2+^] are observed for all GECI proteins and their mutants providing evidence for the existence of conformational changes contributing to the degree of cooperativity and free energy coupling in Ca^2+^.CaM – target interactions. mGCaMP3 effectively switching between low and high fluorescence forms is in contrast with fluorescent indicators e.g. Fluo-3 the Ca^2+^ association rate of which is linearly dependent on [Ca^2+^]^29^ and the recently developed red Ca^2+^ indicator R-GCaMP2 which has reduced cooperativity (*n* = 1.2)^31^.

Interestingly, the limiting rates for GCaMP3 and GCaMP3_*bright*_ show no temperature dependence in the 20 °C to 37 °C range. The GCaMP6f probe behaves the same way (N. Helassa and K. Török, unpublished data). The lack of temperature dependence suggests a diffusion limited binding process. As the Ca^2+^ binding rates are unlikely to be limiting, it may be that peptide binding within the GCaMP construct is sterically constrained, thus the limiting rate is set by the low frequency of collision. In contrast, GCaMP3_*fast*_ displayed a 5-fold increase of the saturating rate over the same temperature range. By contrast, dissociation rates of all the probes were greater at 37 °C than at 20 °C, an advantageous property for *in vivo* applications.

Taking advantage of our detailed measurements of Ca^2+^ association and dissociation kinetics, we developed a kinetic model to quantitatively account for the observed behaviour of GCaMP3_*fast*_ (Fig. 1c). The model comprised the minimum number of steps required to take into account the Ca^2+^ binding properties of the CaM EF-3 mutant, its interactions with the RS20 target peptide and the processes that lead to restoration of fluorescence emission by cpEGFP^4^,^19^,^20^,^32^,^33^. Although Ca^2+^ association rate constants in the presence of a target peptide have not been determined, as a first approximation we used the same or similar rate constants as reported for Ca^2+^ binding to CaM^32^. A first intermediate had Ca^2+^ bound to the N-lobe sites and formed a partial complex (with relative fluorescence *F*_1_) by peptide binding to the P1 CaM binding site; a second intermediate (with relative fluorescence *F*_2_) formed by completing Ca^2+^ binding to the C-lobe site and peptide at the P2 site too. Postulation of two fluorescent conformers was based on the observation of biphasic Ca^2+^ association and dissociation kinetics for some mGCaMP and the requirement for functional N-lobe Ca^2+^ binding sites in CaM (EF-1 and EF-2) for fluorescence to develop.

Cooperativity was expressed by intrinsic rate constants in the model and was reflected by one or both fluorescent isomers being formed by essentially irreversible processes (Fig. 1c). The very small reverse rate constants determined in our best-fit analysis (Supplementary Table S3) were supported by experimental evidence showing slow peptide dissociation at 0.0075 s^−1^ from the Ca^2+^.CaM.peptide complex with an RS20 analogue peptide (Trp peptide)^19^. As a consequence, peptide release rates were modelled to be determined by the Ca^2+^ dissociation rates from the Ca^2+^.CaM.cpEGFP.RS20 mutant complexes (Supplementary Fig. S13). As a further constraint, the overall *K*_d_ was kept close to the measured value. The same model is applicable to GCaMP3_*bright*_ by omission of the red star signifying the RS-1 mutation (Fig. 1c). The model, appropriately taking into account the mutated sites, was applied to GCaMP3 and a selection of mGCaMP3 (Supplementary Materials, Supplementary Fig. S13, S14 and Supplementary Table S3) for which the kinetic data are overlaid with fitted curves (Fig. 2b,c,f,h, Supplementary Fig. S4–S6 and Supplementary Table S3).

The new data and interpretations presented here show that we have made a systematic and successful effort to improve the kinetics of biologically engineered calmodulin-based fluorescent peptide Ca^2+^ indicators (GECI). This further suggests a strategy for improving the fluorescence and kinetic properties of GCaMP-type GECI. N-lobe CaM mutations are detrimental whereas C-lobe mutations improve or are neutral to 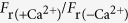. Ca^2+^ kinetics are most markedly affected by combining mutations of the RS20 peptide and CaM EF-hands. The specific mutations carried out on GCaMP3 and derived GECI are applicable to the new generations of GECI e.g. GCaMP5, GCaMP6 and R-CaMP2^13^,^15^,^31^ for generating fast-response variants. Two-photon absorption measurements show that recordings with deep penetration in GECI-expressing transgenic animals and tissues from these probes will be possible. Furthermore, the probes can be targeted to specific cell types, organelles and proteins using different promoters, targeting sequences and binding domains. Thus, given the potentials and limitations of genetically engineered Ca^2+^ probes, a major hurdle has been overcome.

## Methods

### Materials

GCaMP6f, G-GECO 1.0 and GEM-GECO plasmids were purchased from Addgene (plasmid #40755, #32466 and #32463, respectively). pET41a and pEGFP-N1 vectors were obtained from Novagen and Clontech, respectively. XL10-Gold and BL21 (DE3) Gold cells were purchased from Invitrogen. Fluo-3 was purchased from Molecular Probes and EZ-Run protein ladder from Fisher Bioreagents. Restriction enzymes were obtained from New England Biolabs and T4 DNA ligase from Fermentas. Br_2_-BAPTA was purchased from Life Technologies.

### Cloning of GECIs into bacterial GST-expression vector

The *gcamp3* gene was subcloned by restriction-ligation from pUC57 into pET41a using BglII and NotI restriction enzymes and T4 DNA ligase following manufacturers’ protocols. The *ggeco* and *gemgeco* genes contained in pTorPE vectors^11^ were subcloned into pET41a as described above except using NcoI and HindIII restriction sites. The resulting plasmids express GST-fused proteins.

### Site-directed mutagenesis of GECIs

A series of DNA mutations were performed on pET41a-GCaMP3, pET41a-G-GECO and pET41a-GEM-GECO. Site-directed mutagenesis was carried out following the QuickChange II XL protocol (Agilent Technologies) to mutate the CaM EF-hands using the following primers (5′→3′): EF-1, GCTTTCTCCCTATTTGCCAAGGACGGGGATGGG; EF-2, CATGATCAATGAAGTAGCTGCCGACGGTAATGGC; EF-3, GCGTTCCGTGTGTTTGCTAAGGATGGCAATGGC; EF-4, GATCAGGGAAGCAGCCATCGATGGGGATGG; for the Trp →Tyr substitution in the RS20 peptide sequence, primer RS-1 (5′ →3′), CGACTCATCACGTCGTAAGTACAATAAGACAGGTCACGCAG was used. Mutations were confirmed by DNA sequencing (Beckman Coulter Genomics).

### Expression and purification of GECI proteins

GCaMP3, G-GECO and GEM-GECO proteins were overexpressed in *E. coli* BL21(DE3) Gold cells. Cells were grown at 37 °C and expression was induced overnight at 20 °C in the presence of 0.5 mM isopropyl thio-β-D-galactoside (IPTG). Cells were resuspended in 50 mM Na^+^-HEPES, 200 mM NaCl, pH 7.5 containing one tablet of Complete protease inhibitor cocktail (Roche, Basel, Switzerland). Cell lysis was performed by sonication on ice (VibraCell, Jencons PLS) and clarified lysates were purified by a single-step GST-chromatography (GSTrap, ÄKTA Purifier, GE Healthcare) at 4 °C. The column was equilibrated with 50 mM Na^+^-HEPES, 200 mM NaCl, pH 7.5. The purified protein was eluted in 50 mM Na^+^-HEPES, 200 mM NaCl, 10 mM reduced glutathione, pH 7.5 and aliquoted fractions were stored at −80 °C. Purity of the eluted fractions was determined by SDS-PAGE (gradient of 6.4%–20% acrylamide/bisacrylamide) and the identity of the GCaMP3, G-GECO and GEM-GECO proteins was confirmed by MALDI mass spectroscopy using sinapinic acid as a matrix (BruckerUltraFlex III, SGUL Biomics Centre).

### Measurement of protein concentration

Mutant GCaMP3 proteins were highly purified (Supplementary Fig. S1), allowing protein concentration to be determined spectroscopically. The absorption spectra of all GECI and mGECI comprised three peaks at wavelengths 280 nm, 400 nm and 500 nm. Protein concentrations were determined with molar extinction coefficients (*ε*_o_) at 280 nm calculated from the amino acid composition^34^ using a Nanodrop 1000 spectrophotometer (Thermo Scientific). The spectroscopically determined protein concentrations using the calculated ε_o(280)_ were verified by parallel measurements using the Bradford assay^35^. We observed that the absorption ratio A_280_/A_400_ was affected by the mutations introduced. From the ε_o(280)_ values and the A_280_/A_400_ ratios, the ε_o(400)_ values were calculated for each probe in a folded state at physiological pH rather than following protein denaturation^11^ (Supplementary Table S4).

The stability of GCaMP3_*fast*_ was tested at 37 °C by measurement of fluorescence emission over 4 h. Excitation wavelength was 492 nm.

The GST-tag that was introduced on the N terminus for purification purposes had no effect on the fluorescence properties. The *K*_d_ for Ca^2+^ of GST-GCaMP3 was 0.33 ± 0.01 μM compared to 0.42 ± 0.06 μM of GCaMP3 from which the GST-tag had been removed on the column by thrombin digestion (Supplementary Fig. S12).

### Determination of free Ca^2+^ concentrations ([Ca^2+^])

[Ca^2+^] were calculated using the two-chelators Maxchelator program^36^ and verified by titrating Fluo-3 in 50 mM K^+^-HEPES, 100 mM KCl, 2 mM MgCl_2_, 10 mM EGTA pH 7.5 as described below. Data were fitted to a one-site specific binding equation using Prism GraphPad 6 software, giving a dissociation constant for Fluo-3 for Ca^2+^ (*K*_d_) of 326 ± 12 nM. Similar dissociation constant (*K*_d_ = 325 nM) has been reported by Molecular Probes. Fluo-3 was used as a reference compound to verify the calculated [Ca^2+^] of the solutions made for the stopped-flow experiments of association kinetics (Supplementary Fig. S12).

### Equilibrium Ca^2+^ binding

Ca^2+^ affinity assays of Fluo-3, GECI and mGECI were performed by continuous titration using an automated syringe pump (ALADDIN 1000, WPI). Fluo-3 or GECI proteins at 50–100 nM concentration (50 mM K^+^-HEPES, 100 mM KCl, 2 mM MgCl_2_, 5 mM EGTA, pH 7.5 at 20 °C) were titrated with 325 mM CaCl_2_ at a 10 μL/min flow rate in a stirred 3 mL cuvette. Fluorescence was measured at 492 nm excitation and 512 nm emission wavelengths using a Fluorolog3 spectrofluorimeter (Horiba Scientific). For mGCaMP3 RS-1 EF-hand mutants, Br_2_-BAPTA was used for Ca^2+^ buffering instead of EGTA. Fluorescence records were corrected for dilution and photobleaching. Data were normalised and expressed as bound fraction and Ca^2+^ dissociation constant (*K*_d_) and cooperativity (*n*) were obtained by fitting the data to the Hill equation using Prism GraphPad 6 software. All titrations were performed at least in triplicates and expressed as mean ± s.e.m.

### Stopped-flow fluorimetry

Ca^2+^ association and dissociation kinetic experiments of GCaMP3, G-GECO and GEM-GECO proteins and of Fluo-3 were carried out on a Hi-Tech Scientific SF-61DX2 stopped-flow system at 20 °C, unless specified otherwise. Fluorescence excitation was set to 492 nm for mGCaMP3 and mG-GECO, or 392 nm for mGEM-GECO. Fluorescence emission was collected using a 530 nm cut-off filter. At least 3 replicates were averaged for analysis. Data were fitted to either a single or a double exponential to obtain the rise or decay rate using KinetAsyst software (TgK Scientific). As the “dead-time” of the instrument (time between the mixing and the point of observation) is ~1.5 ms, rates above 500 s^−1^ (*t*_1/2_ of 1.4 ms) cannot be accurately measured. To overcome this limitation, kinetics were measured at lower temperatures and the rates obtained by extrapolation from Arrhenius plots.

#### Association kinetics

The solution containing 1 μM protein in 50 mM K^+^-HEPES, 100 mM KCl, 2 mM MgCl_2_, 10 mM EGTA pH 7.5 was rapidly mixed (1:1) with 50 mM K^+^-HEPES, 100 mM KCl, 2 mM MgCl_2_, 10 mM EGTA pH 7.5 containing increasing [Ca^2+^] concentrations (concentrations in the mixing chamber). For the determination of temperature dependence of Ca^2+^ association rates, protein samples at 1 μM concentration were mixed as above to give a 20 μM final [Ca^2+^] in the mixing chamber. The Ca^2+^ association rates to Fluo-3 were too fast to measure.

#### Dissociation kinetics

The solution containing 1 μM protein in 50 mM K^+^-HEPES, 100 mM KCl, 2 mM MgCl_2_, pH 7.5 with saturating Ca^2+^ between 0.1–0.5 mM was rapidly mixed (1:1) with 50 mM K^+^-HEPES, 100 mM KCl, 2 mM MgCl_2_, 10 mM EGTA pH 7.5 (concentrations in the mixing chamber). The Ca^2+^ dissociation rate from Fluo-3 was measured using the same conditions.

### Quantum yield determination

The concentration of GCaMP3 proteins was adjusted such that the absorbance at the excitation wavelength (490 nm) was between 0.001 and 0.1. A series of dilutions was prepared in a buffered solution (50 mM HEPES, pH 7.5, 100 mM KCl, 2 mM MgCl_2_ with either 5 mM EGTA or 1 mM CaCl_2_) and the fluorescence spectra were recorded on a Fluorolog3 (Horiba Scientific). GCaMP3 quantum yield measured in Ca^2+^-saturated buffer was used as a reference (Φ_+Ca_^2+^ = 0.44^11^). Data were plotted as integrated fluorescence intensity as a function of absorbance and fitted to a linear regression(S). Quantum yields were obtained using the following equation: Φ_protein_ = Φ_GCaMP3_ × (S_protein_/S_GCaMP3_).

### pH sensitivity of GCaMP3 proteins

To determine the apparent p*K*a for each GCaMP3 proteins, a series of buffers were prepared. Depending on their respective pH buffering range, appropriate buffer was used for the measurements (MES for pH 6–6.5, HEPES for pH 7–8, TRIS for pH 8.5–9 and CAPS for pH 10). The pH titrations were performed by recording fluorescence spectra in Ca^2+^-free (50 mM buffer, 100 mM KCl, 2 mM MgCl_2_, 2 mM BAPTA) or Ca^2+^-saturated (50 mM buffer, 100 mM KCl, 2 mM MgCl_2_,1 mM CaCl_2_) using 1 μM protein in 0.5 pH unit intervals (Fluorolog3, Horiba). BAPTA was chosen as Ca^2+^ chelator because of its stable affinity for Ca^2+^ over the pH range.

### Cloning of mGECIs into a mammalian expression vector

For expression in eukaryotic cells, the GCaMP3, G-GECO and GEM-GECO mutant DNAs were subcloned from pET41a into pEGFP-N1 vectors by restriction-ligation using BglII and NotI restriction enzymes and T4 DNA ligase following manufacturer’s recommendations. During this process, the *gfp* initially present in the pEGFP-N1 vector was replaced by the *gcamp3, ggeco* or *gem-geco* gene.

### Adenovirus generation

Adenoviruses (Ad5 backbone) expressing GCaMP3, GCaMP6f and GCaMP3_*fast*_ under the control of the CMV promoter were obtained from Vector Biolabs (USA). Titer was estimated to be 5 × 10^10^ PFU/mL.

### Neonatal cardiomyocyte (rN-CM) isolation

Rat neonatal CMs were isolated using an isolation kit from Worthington Biochemical Corporation (Lakewood, NJ 08701). One to three day-old neonatal rats were decapitated and the beating hearts were surgically removed and placed in chilled Hank’s Balanced Salt Solution (HBSS). The main vessels and atria were removed and the ventricles were minced with a razor blade to pieces <1 mm^3^ that were incubated in HBSS with trypsin (50 μg/mL) for 14–16 h at 4 °C. The digestion was then arrested by exposure to trypsin inhibitor (200 μg/mL) for 20 min in 37 °C. Thereafter collagenase (100 U/mL) was used for 30 min to isolate single rN-CM, which were filtered through a cell strainer and centrifuged at 1000 rpm for 3 min. Cells were re-suspended in Dulbecco’s Modified Eagle’s Medium (DMEM) containing 10% fetal bovine serum (FBS) with 1% penicillin/streptomycin and 1% non-essential amino acids, plated on 100 mm dishes and placed in the incubator for 1–2 h to eliminate fibroblasts. Isolated single rN-CM were plated onto non-treated glass cover slips and used for adenovirus infection with Ad-GCaMP3, Ad-GCaMP6f or Ad-GCaMP3_*fast*_. After adenoviral infection of neonatal cardiomyocytes, the level of expression changes several-fold during 2–5 days of experimentation without significant changes in the time course of the measured fluorescence transients. Cells were used for confocal imaging and electrophysiology 48–72 h after viral infection. Although contraction artefacts are not particularly bothersome in short time cultured neonatal cardiomyocytes, they were suppressed by treating the cells with 10 μM blebistatin.

### Caffeine stimulation of cardiomyocytes

Rapid applications of 3 mM caffeine were used in the external K^+^-free solutions to probe the cellular Ca^2+^ stores or spontaneous Ca^2+^ release from sarcoplasmic reticulum.

### Voltage clamp procedures on cardiomyocytes

Cells were voltage clamped in the whole-cell configuration at −50 mV to measure voltage dependent I_Ca_ using a Dagan amplifier and pClamp software (Clampex 10.2). In most experiments the membrane under the patch pipette was ruptured and the cells dialyzed with Ca^2+^-buffered pipette solutions containing 110 mM Cs_+_-aspartate, 5 mM NaCl, 20 mM TEA-Cl, 5 mM Mg^2+^-ATP, 0.1 mM EGTA 0.1 mM CaCl_2_, 10 mM glucose and 10 mM Cs^+^-HEPES pH 7.2. In some experiments the cells were examined with the perforated patch technique using pipette solutions containing 145 mM Cs^+^-glutamate, 9 mM NaCl, 1 mM MgCl_2_, 10 mM Cs^+^-HEPES pH 7.2 and 0.69 mg/mL amphotericin B (Fisher Scientific). The extracellular solution used during experiments contained 137 mM NaCl, 5.4 mM KCl, 2 mM CaCl_2_, 1 mM MgCl_2_, 10 mM glucose and 10 mM Na^+^-HEPES pH 7.4.

### Two-dimensional confocal imaging

Cytosolic Ca^2+^, [Ca^2+^]_i_, was measured simultaneously with the patch clamp or independently using a Noran Odyssey XL rapid two-dimensional laser scanning confocal microscopy system (Noran Instruments, Madison, WI, USA) attached to a Zeiss Axiovert TV135 inverted microscope fitted with an ×63 water-immersion objective lens. The excitation wavelength of the argon ion laser was set to 488 nm, and fluorescence emission was measured at wavelengths >515 nm. Cells were imaged confocally at 30–120 frames/s depending on the experimental requirement. To reduce photobleaching, the laser beam was electronically shuttered and triggered to open on command by the pClamp program only during acquisition of data. Images were filtered by 2 × 2 pixel-averaging. Results are presented as whole-cell measurements by integrating the fluorescence response from an entire cell (Fig. 3), or as local responses from regions of interest (ROI), as detailed in legend for Supplementary Fig. S9. The amplitudes of the Ca^2+^-dependent cellular fluorescence signals were quantified as Δ*F*/*F*_0_. Experiments were carried out at room temperature (22–24 °C).

### Cell culture, transfection and live cell imaging of Fura-2 and GCaMP fluorescence in human umbilical vein endothelial cells

HUVEC were grown and transfected as previously described^37^,^38^. For live cell imaging Nucleofected HUVEC were seeded onto 35 mm diameter poly-D-lysine coated glass bottomed culture dishes (MatTeK, Ashland, MA) and used 24 h post Nucleofection. Cells were transferred to physiological saline solution (140 mM NaCl, 5 mM KCl, 1.8 mM CaCl_2_, 10 mM glucose, 10 mM Na^+^-HEPES pH 7.4) and placed on the stage of an inverted Olympus IX71 fluorescence microscope. The microscope was equipped with an Olympus UPLSAPO ×100 1.40 NA objective and an OptoLED light source (Cairn Research, Faversham, UK) for fast epifluorescence illumination of Fura-2 (355 ± 10 nm and 380 ± 11 nm) and EGFP (470 ± 20 nm) fluorescence. Excitation light was directed to the specimen via a 500DCXR dichroic mirror (Chroma Rockingham, USA) and emitted light collected through a 535 ± 30 nm emission filter onto an Ixon3 EMCCD camera (Andor, Belfast, UK) operated in frame transfer mode at full gain and cooled to −68 °C. Images were acquired at 30 frames/s using Winfluor software (http://spider.science.strath.ac.uk).

Fura-2 loading was performed under conditions where it had previously been established that no perturbation of stimulated exocytosis, a particularly sensitive Ca^2+^-dependent cell output, of fluorescent secretory granules occurred^37^,^38^. For stimulation GCaMP_*fast*_-expressing cells with low expression levels (low intrinsic levels of resting fluorescence) were chosen to minimise the effect of buffering, and the time courses for GCaMP_*fast*_ signals were similar with or without simultaneous Fura-2 loading. Arbitrary units of fluorescence were used as Fura-2 calibration is not meaningful due to the saturating levels of [Ca^2+^] rise.

To determine the dynamic range of mGECI, cells were stimulated using ionomycin (2 μM) to increase intracellular free [Ca^2+^] to high levels. For physiological studies cells were stimulated with the extracellular hormone ATP (2 μM). Drug application was by pressure injection from a glass pipette (Warner instruments, Kent, UK) at ~2 p.s.i. positioned close to the cell as previously described^38^. Image analysis was carried out in Winfluor. Dataset plotting, fitting and analysis were performed in Microcal Origin 7.5 (OriginLab Corporation, Northampton, MA). Results are expressed as mean ± s.e.m. unless indicated otherwise.

To determine the time course for cytoplasmic Rura-2 and/or GCaMP fluorescence changes during ATP stimulation a rectangular region of interest (ROI) was placed over a peripheral region of each cell, excluding the nucleus and immediate perinuclear region. The mean background fluorescence was measured by ROI with the same dimensions placed at a point at which no cell was present. GCaMP fluorescence changes were obtained from the background-subtracted fluorescence at 470 nm excitation within the WinFluor program. Fura-2 ratio changes were calculated by dividing the background-subtracted fluorescence at 355 nm by that at 380 nm in WinFluor. The ratio of the resulting background-subtracted fluorescence signals at 355 nm and 380 nm were taken to represent the change in [Ca^2+^]_i_, as previously described^38^.

### Cell culture, transfection and live cell imaging of Fura-2 and GCaMP fluorescence in HEK293T cells

HEK293T cells were grown on a 100 mm × 20 mm culture dish in DMEM supplemented with 10% fetal bovine serum and 1% Penicillin-Streptomycin solution. At 90% confluence cells were plated on to glass coverslips (12 mm diameter) in 35 × 10 mm petri dishes. Transfection was accomplished using Lipofectamine™ 2000 (Invitrogen): 0.5 μg of plasmid DNA was combined with 8 μL Lipofectamine™2000 reagent in 200 μL Opti-MEM, this was then added to the cell culture medium. The transfection medium was removed after 8–10 hours and experiments were performed 48 hours after transfection.

Transfected HEK293T cells were loaded with 1 μM Fura-2 AM (Molecular Probes, Eugene, OR, USA) for 20 minutes at 37 °C, and then incubated in dye-free solution for at least 20 minutes before beginning experiments to allow for the de-esterification. Cells were mounted on the stage of an inverted Nikon microscope with a ×40 oil-immersion objective and perfused with a Tyrode’s solution at 37 ± 2 °C (137 mM NaCl, 10 mM HEPES, 10 mM D-glucose, 5.4 mM KCl, 2 mM CaCl_2_, 1 mM MgCl_2_). Ca^2+^ release in HEK293T cells was induced by a 5 s pulse of 100 μM ATP produced by a digitally triggered solution ‘puffer’ positioned ~250 μm away from cell. A rapid (1.2 kHz) scanning dual beam excitation fluorescence photometry apparatus (Vibraspec Inc., Bear Island, ME, USA) was used to simultaneously monitor whole-cell response to changes in [Ca^2+^] by Fura-2 and GCaMP3 or mGCaMP3 by employing alternating 380/25 and 488/20 nm wavelengths, respectively^39^.

### Measurement of two-photon cross-sections

Two-photon absorption (TPA) or two-photon cross-section (σ_2_) is the absorption coefficient for the simultaneous absorption of two photons for the excitation of a fluorophore. TPA becomes relevant at high light intensities and as it is generated by the sum of the energies of two photons, it occurs at approximately double of the wavelength at which one photon excitation would have occurred. The two-photon fluorescence cross-sections of GCaMP3, G-GECO, GEM-GECO and their mutants were typically measured at 940 nm (unless specified otherwise) in 50 mM K^+^-HEPES, 200 mM NaCl, pH 7.5, 10 mM reduced glutathione at high Ca^2+^ (200 μM) or low Ca^2+^ (2 mM EGTA). Measurements were made in a 50 μL quartz cuvette with 3 mm path length. Excitation was with femtosecond-pulsed light from a tuneable TiS laser (710–990 nm, 100–150 fs pulses 80 MHz; MaiTaiBB, Spectra Physics) focused into the cuvette with a 50 mm f.l. achromatic doublet after 3× expansion. Average power was monitored after the cuvette. Fluorescence was measured normal to the excitation path through a band pass filter (Chroma ET525/50-2P) with a photodiode and I/V amplifier. A reference spectrum for fluorescein (20 μM in 10 mM Na^+^-borate buffer pH 11) was determined in each run. After subtraction of background, fluorescence cross-sections were calculated for Ca^2+^-saturated and Ca^2+^-free protein with reference to the published values for fluorescein^23^.

### Data analysis, statistics and kinetic modelling

Biophysical experiments were performed at least in triplicates and analysed using GraphPad Prism and KinetAsyst (tgk Scientific) software. Cardiac myocytes, HUVEC and HEK293T cell experiments were carried out on three independent preparations each. The total number of cells analysed in each condition is given in the figure legends. Significance level was obtained using the one-way ANOVA test with multiple comparisons. P values in the figures are represented by stars (*P < 0.05, **P < 0.01, ***P < 0.001, ****P < 0.0001). Kinetic modelling was performed using DynaFit4 software according to the scheme presented in Supplementary Fig. S14.

## Additional Information

**How to cite this article**: Helassa, N. *et al.* Fast-Response Calmodulin-Based Fluorescent Indicators Reveal Rapid Intracellular Calcium Dynamics. *Sci. Rep.* **5**, 15978; doi: 10.1038/srep15978 (2015).

## Supplementary Material

Supplementary Information

## Figures and Tables

**Table 1:** Summary of the biophysical characteristics of GCaMP3_*fast*_ and GCaMP3_*bright*_ probes.

Protein	*F*_r_		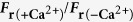	*K*_d_ (μM)	*n*	*k*_on(lim)_ (s^−1^)		*k*_off_ (s^−1^)		σ_2_ (10^−50 ^cm^4^.s.photon^−1^)	
	−Ca^2+^	+Ca^2+^				20 °C	37 °C	20 °C	37 °C	−Ca^2+^	+Ca^2+^
GCaMP3	1.0	6.3	6.3	0.33 ± 0.01	3.2 ± 0.1	20 ± 1	19 ± 1	1.5 ± 0.1	5.3 ± 1	0.06^a^	0.76^a^
mGCaMP3 RS-1 EF-3 (GCaMP3_*fast*_)	0.6	5.1	8.8	2.8 ± 0.3	4.3 ± 0.2	145 ± 2	743^b^	62 ± 2	210 ± 6	0.1	0.47
mGCaMP3 EF-4 (GCaMP3_*bright*_)	0.3	4.2	14	0.93 ± 0.01	4.7 ± 0.1	135 ± 8 (0.5)^c^ 19 ± 1 (0.5)^c^	139 ± 41 (0.5)^c^ 26 ± 2 (0.5)^c^	2.8 ± 0.1	15 ± 0.2	0.22	2.00

Relative fluorescence increase 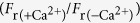, *K*d and Hill coefficient (*n*) values were obtained from the equilibrium Ca^2+^ binding titrations at 20 °C. Fluorescence rise (limiting rate, *k*_on(lim)_) and decay (*k*_off_) rates were measured by Ca^2+^ association and dissociation stopped-flow kinetic experiments both at 20 °C and 37 °C. Two-photon cross-section (σ_2_) values were determined from two-photon excitation spectra at 940 nm, unless otherwise specified, using fluorescein as a reference. ^a^Recorded at 930 nm. ^b^Measurements were made at 20, 25 and 30 °C. Rates were too fast to measure at 37 °C, thus value was extrapolated from Arrhenius plot (Fig. 2i). ^c^Biphasic association kinetic records were fitted with two exponentials. The rate of each phase is given with the relative amplitudes in parentheses. Error represents the standard error of the estimate for the average of three records.
