# Supplementary material for: Fast-Response Calmodulin-Based Fluorescent Indicators Reveal Rapid Intracellular Calcium Dynamics
Source: Sci Rep. 2015 Nov 3;5:15978. doi: 10.1038/srep15978 (PMC4630588; doi:10.1038/srep15978)
Supplement: Supplementary Information [file srep15978-s1.pdf]

FAST-RESPONSE CALMODULIN-BASED FLUORESCENT INDICATORS REVEAL RAPID INTRACELLULAR CALCIUM DYNAMICS

Nordine Helassa[a], Xiao-hua Zhang[b], Ianina Conte[a,c], John Scaringi[b], Elric Esposito[d], Jonathan Bradley[d], Thomas Carter[a,c], David Ogden[d], Martin Morad[b] and Katalin Török[a]*

[a]Institute of Cardiovascular and Cell Science, St George's, University of London, London SW17 0RE, UK; [b]Department of Regenerative Medicine and Cell Biology, Medical University of South Carolina, Charleston, SC 29425, USA; [c]MRC National Institute for Medical Research, London NW7 3RJ, UK; [d]Laboratoire de Physiologie Cérébrale, Centre National de la Recherche Scientifique and Université Paris Descartes, 75006 Paris, France.

*Corresponding author: K. Török, Phone: +44 2087255832; Fax: +44 2087253581; ktorok@sgul.ac.uk

Supplementary Materials

Fluorescence properties of mGCaMP3, mG-GECO and mGEM-GECO probes

mGCaMP3 EF-3, mGCaMP3 EF-3:4, mGCaMP3 RS-1 and mGCaMP3 RS-1 EF-4 had similar biophysical characteristics to GCaMP3$_{fast}$ and GCaMP3$_{bright}$ (**Supplementary Table S1**). Mutation of G-GECO and GEM-GECO generally resulted in faster Ca^{2+} response kinetics, however mG-GECO and mGEM-GECO remained considerably slower than mGCaMP3.

The affinity measured for G-GECO (K_d of 630 nM) was decreased for both of mG-GECO EF-3 and mG-GECO EF-4 (K_d of 1.3 and 1.7 μM, respectively). They retained the ~2-fold greater $F_{r(+Ca^{2+})}$ / $F_{r(-Ca^{2+})}$ of G-GECO than that for GCaMP3 *in vitro* and were comparable to GCaMP3 in HUVEC. Thus, mG-GECO EF-3 and mG-GECO EF-4 may be suitable for cellular Ca^{2+} tracking. Under two-photon excitation, mG-GECO EF-1, mG-GECO EF-3 and mG-GECO EF-4 had σ_2 (+Ca^{2+}) / σ_2 (-Ca^{2+}) ratios of 26-, 7- and 13-fold, respectively, greatly increased from 3-fold for G-GECO (**Supplementary Fig. S11, Supplementary Tables S1, S2**). Interestingly, mG-GECO EF-2:3 having an outstanding 1.8 mM K_d (and none in the μM range) for Ca^{2+} may be suitable for measuring extracellular Ca^{2+} concentrations (**Supplementary Fig. S3, Supplementary Table S2**).

mGEM-GECO EF-1, EF-3 and EF-4 had similar ratiometric fluorescence dynamic ranges. mGEM-GECO EF-3 had the highest ratiometric fluorescence dynamic range $((F_{(+Ca^{2+})} / F_{(-Ca^{2+})})_{exc\ 492} / (F_{(+Ca^{2+})} / F_{(-Ca^{2+})})_{exc\ 392}$ of 108, with the mGEM-GECO EF-4 $((F_{(+Ca^{2+})} / F_{(-Ca^{2+})})_{exc\ 492} / (F_{(+Ca^{2+})} / F_{(-Ca^{2+})})_{exc\ 392}$ of 92) and EF-1 mutants following closely $((F_{(+Ca^{2+})} / F_{(-Ca^{2+})})_{exc\ 492} / (F_{(+Ca^{2+})} / F_{(-Ca^{2+})})_{exc\ 392}$ of 96). The EF-1:2 mutation in mGEM-GECO, as in mGCaMP3 and mG-GECO, led to no detectable response to Ca^{2+} binding. In terms of kinetics, mGEM-GECO EF-2:4 had a Ca^{2+} dissociation rate of 8.3 s^{-1}, ~ 100-fold faster than GEM-GECO for which k_{off} was 0.08 s^{-1} at 20 °C. (**Supplementary Fig. S5** and **Supplementary Table S1, S2).** Although the Ca^{2+} decay kinetics of mGEM-GECO EF-2:4 are much slower than those of GCaMP3$_{fast}$, given their ratiometric fluorescence properties, GEM-GECO mutants may become useful for quantitative measurements of Ca^{2+} transients that occur on the scale of seconds[18]. σ_2 (+Ca^{2+})/ σ_2 (-Ca^{2+}) ratios in the 2- to 9-fold range of mGEM-GECO EF-1, EF-2 and EF-4 suggest their likely suitability for two-photon microscopy (**Supplementary Fig. S11** and **Supplementary Tables S1, S2**).

Modelling the kinetics of Ca^{2+}-induced fluorescence changes of mGCaMP

The model (**Fig. 1c, Supplementary Fig. S13, S14** and **Supplementary Table S3**) was constrained in the following ways: K_d was estimated as the root of the product of the K_d values for each step by the measured Hill coefficient giving $K_{d\ overall} = {}^{n}\sqrt{(K_{d1} * K_{d2}\ldots)}$. Although Ca^{2+} dissociation does not follow the reverse association pathway, constrains are offered by parameters of the isomerisations linking the two fluorescent conformers (see **Supplementary Fig. S14**). Previously determined rate constants for Ca^{2+} association for CaM were used[32]. Association kinetic data were simultaneously fitted to records obtained for a series of [Ca^{2+}].

The model has a number of limitations at present. It was necessary to put forward an 8-step model, however, the observed association and dissociation processes were at most biphasic. Although rate constants were available for Ca^{2+} binding to CaM[26], there is currently no evidence to show that they remain the same in the presence of target peptide. Given the accuracy of [Ca^{2+}] measurements, fitted [Ca^{2+}] concentrations in the nM range were accepted within ± 15% of those measured. The equilibrium of protonated dark and unprotonated fluorescent forms is not known, thus the protein concentration estimate and the associated F_r values were floated to be best fit. At present there is no physical evidence for the fitted rates and it is thus not possible to predict the effect of mutations on specific kinetic constants. This is underlined by the lack of a clear pattern in the assignation of large or small rate constants to specific processes of the model. For example, while the rate constant for the dissociation of Ca^{2+} bound to EF-hand 1, k_{-2} was best fit with values > 1000 s^{-1} for GCaMP3 and the EF-3 and EF-4 mutants, the same process was fit with values < 5 s^{-1} for the EF-3:4 and the RS-1 mutants. To comply with the constraint of the K_d values, this reduction was counterweighted by an appropriately greater value for dissociation rate constant, k_{-2}, of the first peptide binding event (for further detail, see Footnote for **Supplementary Table S3**). These limitations may be overcome by further resolving specific processes.

Supplementary Figure S1

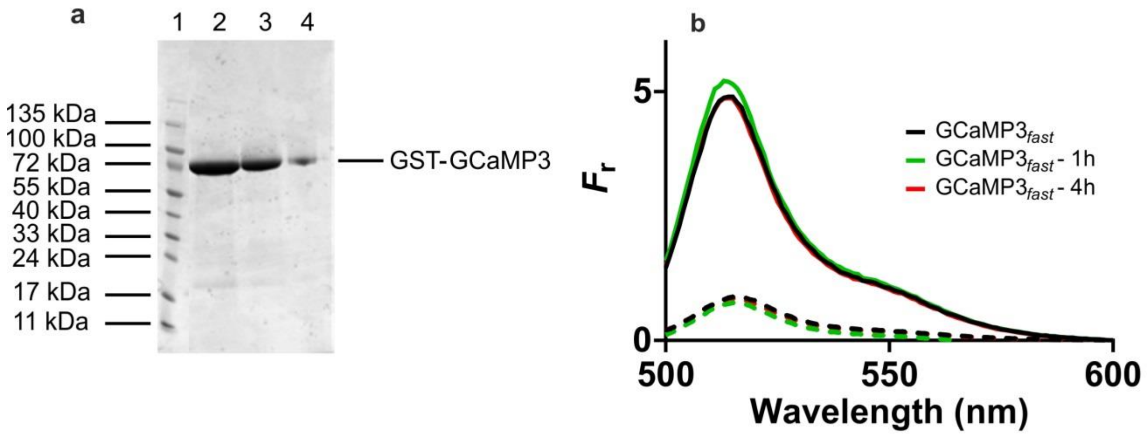

Supplementary Figure S1. Purity and stability of GCaMP3 and mGCaMP3. (**a**) Typical Coomassie Brilliant Blue-stained gradient SDS-PAGE analysis of GCaMP3, following GST chromatography. Lane 1: EZ-Run protein ladder, Lane 2-4: 30 μg, 15 μg and 3 μg of GCaMP3 from eluted fractions, respectively. (**b**) Stability of GCaMP3$_{fast}$ at 37 ^{o}C. GCaMP3$_{fast}$ was incubated at 37 ^{o}C and fluorescence emission spectra of the samples were recorded at 0 h (▬), 1 h (▬) and 4 h (▬). Excitation was at 492 nm. Dashed lines show sample in low Ca^{2+} and solid lines represent sample in high Ca^{2+}.

Supplementary Figure S2

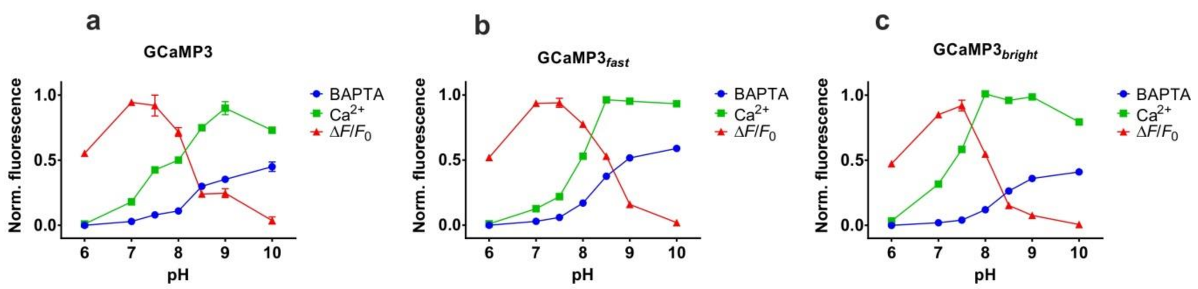

Supplementary Figure S2. pH sensitivity and pK_a determination of (a) GCaMP3; (b) GCaMP3$_{fast}$ and (c) GCaMP3$_{bright}$. Normalised fluorescence in the presence of 1 mM Ca^{2+} (■) and in 2 mM BAPTA (●); $\Delta F/F_0$ (▲).

Supplementary Figure S3

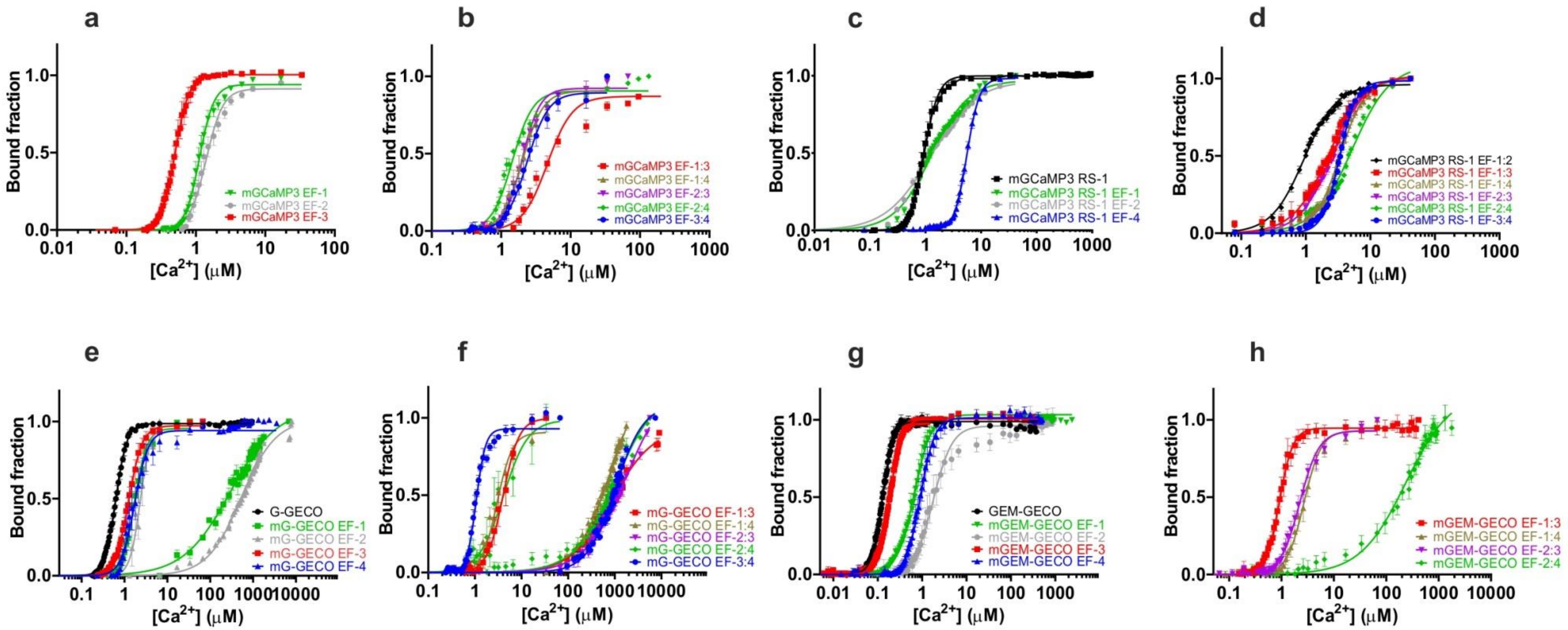

Supplementary Figure S3. Equilibrium Ca^{2+} titrations of mGECIs. (**a**) mGCaMP3 EF-1 (▼), EF-2 (●) and EF-3 (■); (**b**) mGCaMP3 EF-1:3 (■), EF-1:4 (▲), EF-2:3 (▼), EF-2:4 (♦) and EF-3:4 (●); (**c**) mGCaMP3 RS-1 (■), RS-1 EF-1 (▼), RS-1 EF-2 (●) and RS-1 EF-4 (▲); (**d**) mGCaMP3 RS-1 EF-1:2 (♦), RS-1 EF-1:3 (■), RS-1 EF-1:4 (▲), RS-1 EF-2:3 (▼), RS-1 EF-2:4 (♦) and EF-3:4 (●); (**e**) G-GECO (●), mG-GECO EF-1 (▼), EF-2 (●), EF-3 (■) and EF-4 (▲); (**f**) GEM-GECO (●), mG-GECO EF-1:3 (■), EF-1:4 (▲), EF-2:3 (▼), EF-2:4 (♦) and EF-3:4 (●); (**g**) mGEM-GECO EF-1 (▼), EF-2 (●), EF-3 (■) and EF-4 (▲); (**h**) mGEM-GECO EF-1:3 (■), EF-1:4 (▲), EF-2:3 (▼) and EF-2:4 (♦). Fluorescence changes are normalised to F_0 of 0 and F_{max} of 1 and fitted to the Hill equation. Fitted curves are represented by solid lines overlaying the data.

Supplementary Figure S4

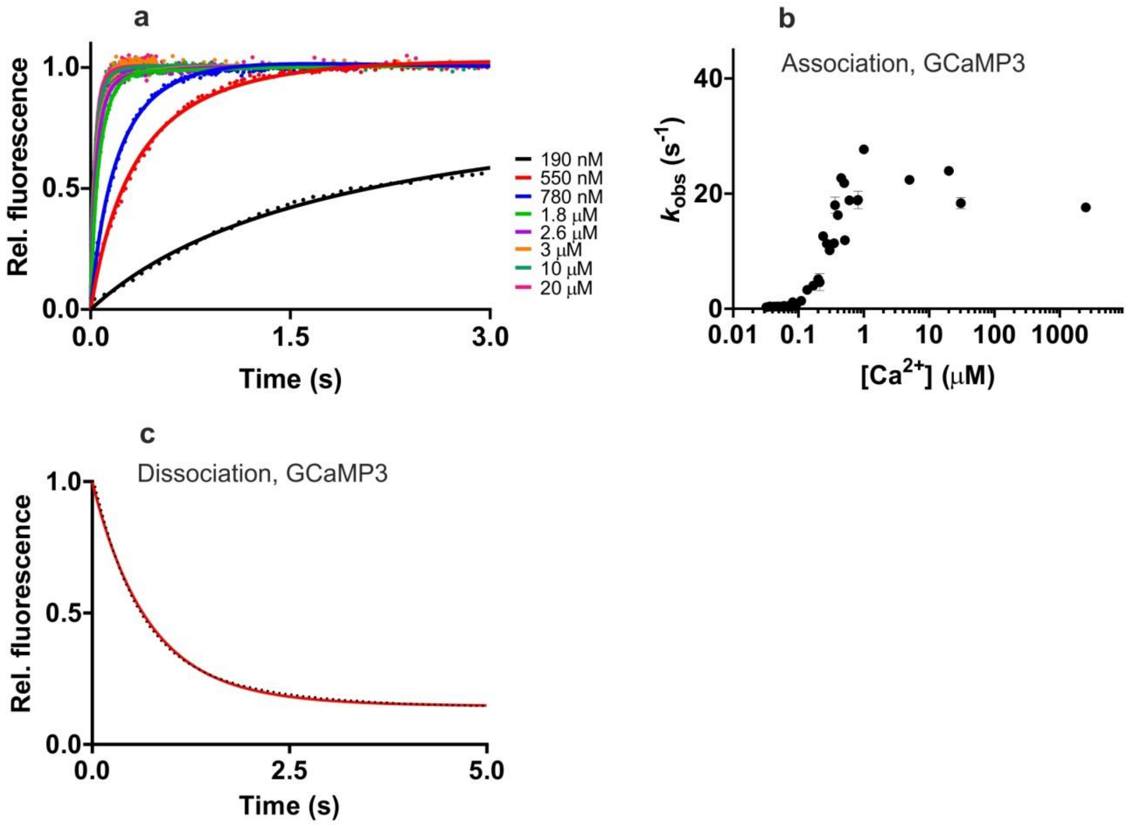

Supplementary Figure S4. Ca^{2+} association and dissociation kinetics with a schematic model of the kinetic mechanism of GCaMP3. (**a**) Association kinetic records were taken at varied final [Ca^{2+}]. Fluorescence changes are normalised to F_0 of 0 and maximum of 1. Experimental data are represented by dotted lines, final [Ca^{2+}] is given in brackets: (···) (190 nM), (···) (550 nM), (···) (780 nM), (···) (1.8 μM), (···) (2.6 μM), (···) (3 μM), (···) (10 μM) and (···) (20 μM). Corresponding solid lines are the fitted curves using parameters shown in **Supplementary Table S3** fitted to the scheme in **Supplementary Fig. S12**. (**b**) Plot of observed rates of Ca^{2+} association with GCaMP3 as a function of final [Ca^{2+}]. (**c**) Dissociation kinetic record of GCaMP3 (···). Fluorescence intensities are relative to the Ca^{2+}-bound form. The solid line (▬) represents the fitted curve with parameters shown **Supplementary Table S3**.

Supplementary Figure S5

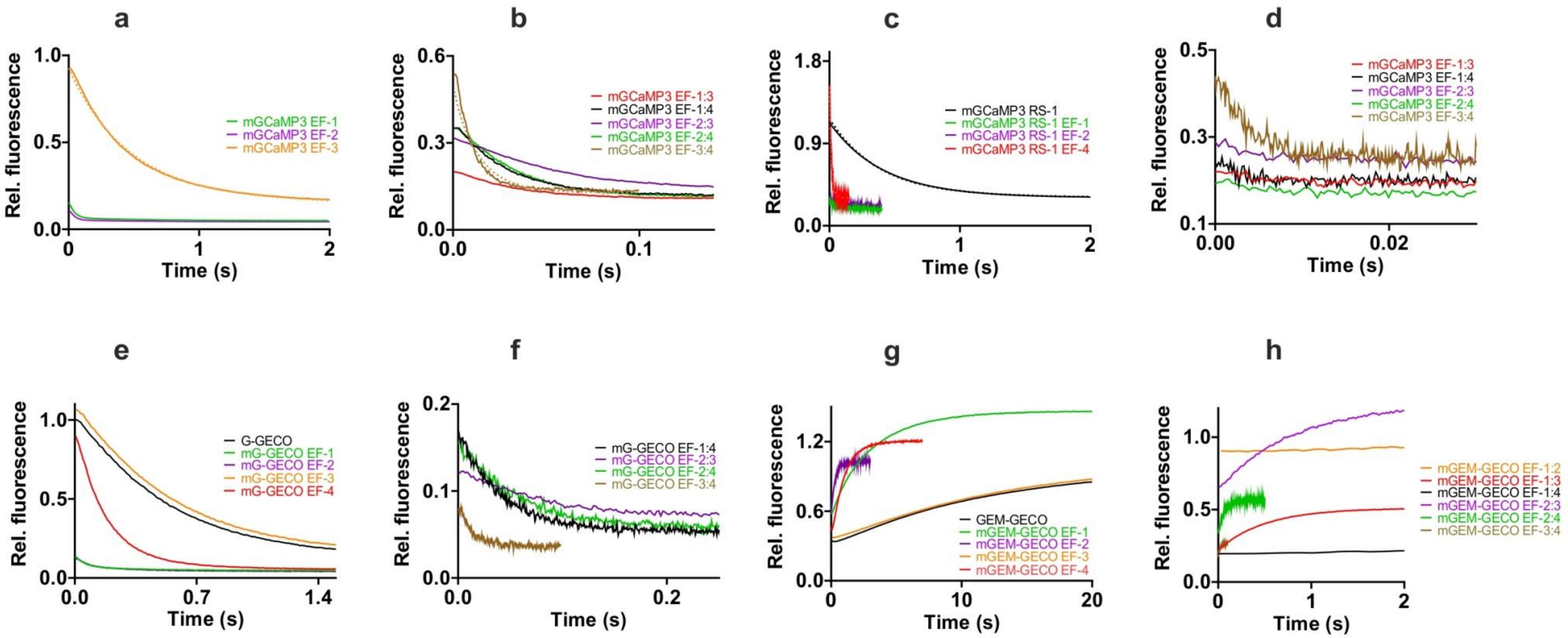

Supplementary Figure S5. Dissociation kinetics for Ca^{2+} of (**a**) mGCaMP3 EF-1 (—), EF-2 (—) and EF-3 (—), overlaid by fitted curve using parameters shown in **Supplementary Table S3**; (**b**) EF-1:3 (—), EF-1:4 (—), EF-2:3 (—), EF-2:4 (—) and EF-3:4 (—), overlaid by fitted curve using parameters shown in **Supplementary Table S3**; (**c**) mGCaMP3 RS-1 (—), RS-1 EF-1 (—), RS-1 EF-2 (—) and RS-1 EF-4 (—), overlaid by fitted curve using parameters shown in **Supplementary Table S3**; (**d**) mGCaMP3 RS-1 EF-1:3 (—), RS-1 EF-1:4 (—), RS-1 EF-2:3 (—), RS-1 EF-2:4 (—) and RS-1 EF-3:4 (—). Fluorescence intensities are relative to Ca^{2+}-bound GCaMP3. (**e**) G-GECO (—), mG-GECO EF-1 (—), EF-2 (—), EF-3 (—) and EF-4 (—), (**f**) mG-GECO EF-1:4 (—), EF-2:3 (—), EF-2:4 (—) and EF-3:4 (—). Fluorescence intensities are relative to Ca^{2+}-bound G-GECO. (**g**) GEM-GECO, (—); mGEM-GECO EF-1 (—), EF-2 (—), EF-3 (—) and EF-4 (—); (**h**) mGEM-GECO EF-1:2 (—), EF-1:3 (—), EF-1:4 (—), EF-2:3 (—), EF-2:4 (—) and EF-3:4 (—). Fluorescence intensities are relative to Ca^{2+}-bound GEM-GECO.

Supplementary Figure S6

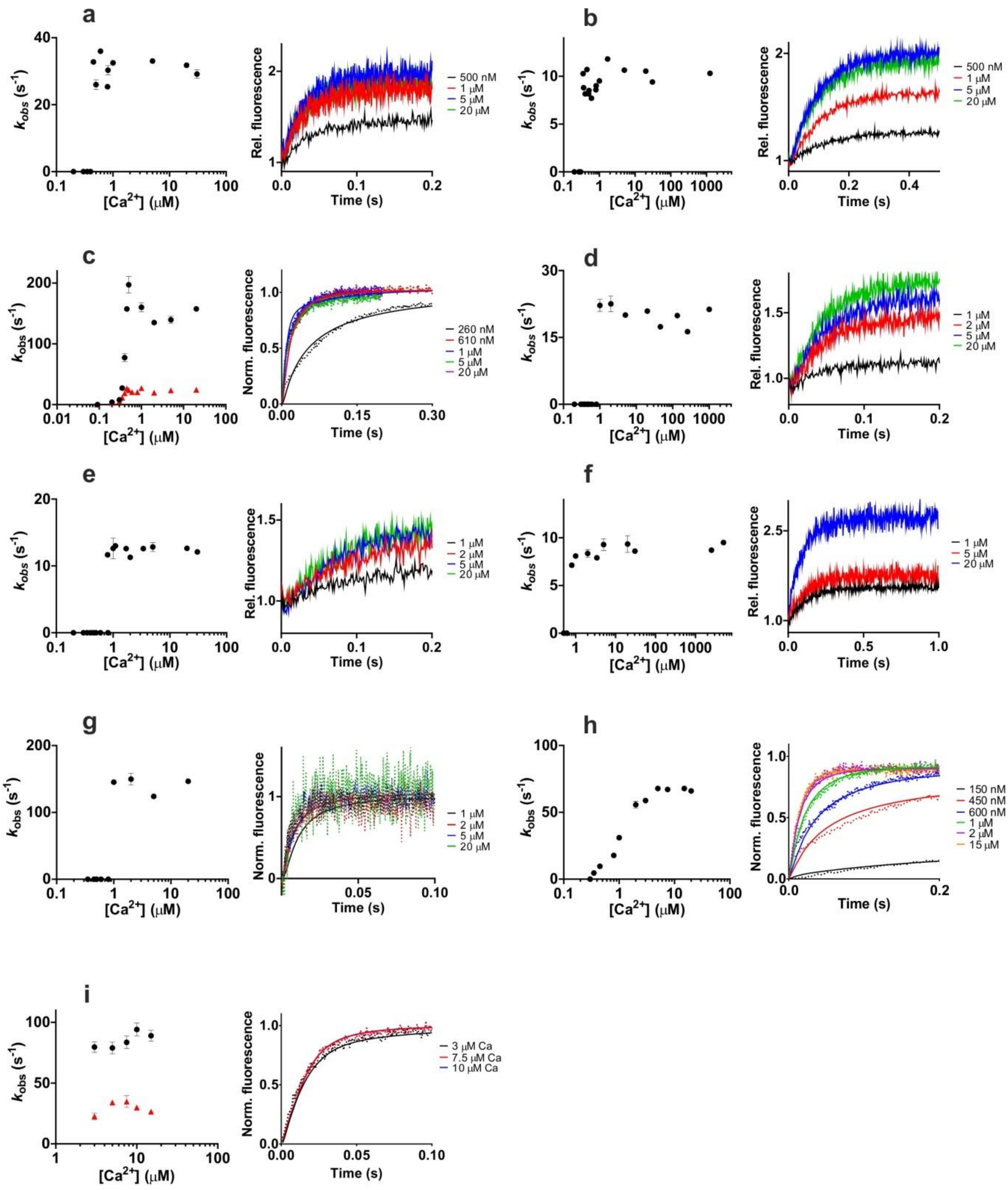

Supplementary Figure S6. Association kinetics for Ca^{2+} of (**a**) mGCaMP3 EF-1, (**b**) mGCaMP3 EF-2, (**c**) mGCaMP3 EF-3, overlaid by fitted curve using parameters shown in **Supplementary Table S3**, (**d**) mGCaMP3 EF-1:4, (**e**) mGCaMP3 EF-2:3, (**f**) mGCaMP3 EF-2:4, (**g**) mGCaMP3 EF-3:4, (**h**) mGCaMP3 RS-1 and (**i**) mGCaMP3 RS-1 EF-4, (**g-i**) experimental records are overlaid with fitted curves using parameters shown in **Supplementary Table S3**. Left panels show plots of the $[Ca^{2+}]$ dependence of the observed rate(s) (k_{obs}), right panels show stopped-flow records at the specified final $[Ca^{2+}]$. Fluorescence changes are normalised to F_0 of 0 and maximum of 1. For mGCaMP3 with biphasic association kinetics, the slow and the fast components are represented in red and black, respectively.

Supplementary Figure S7

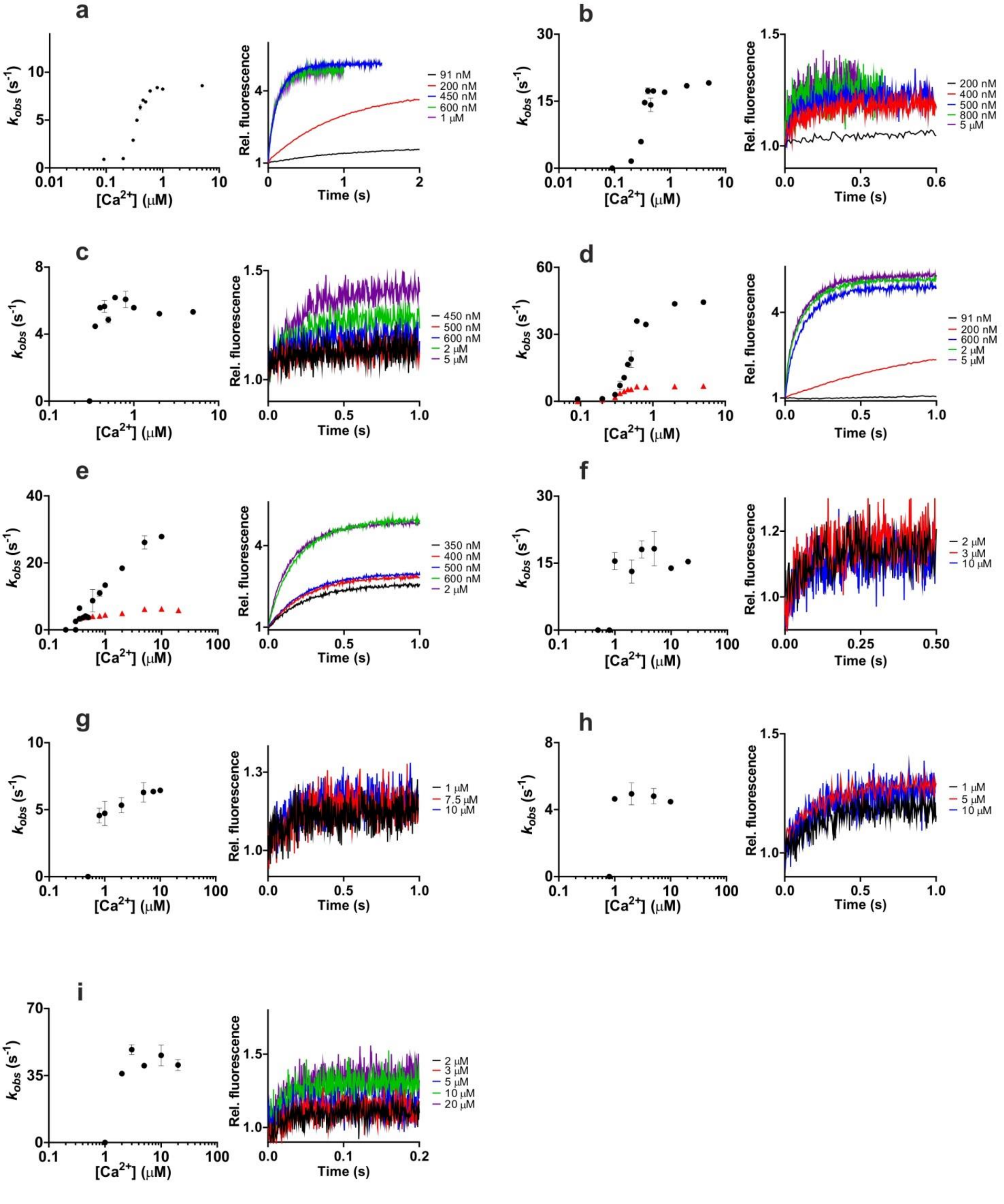

Supplementary Figure S7. Association kinetics for Ca^{2+} of (**a**) G-GECO, (**b**) mG-GECO EF-1, (**c**) mG-GECO EF-2, (**d**) mG-GECO EF-3, (**e**) mG-GECO EF-4, (**f**) mG-GECO EF-1:4, (**g**) mG-GECO EF-2:3, (**h**) mG-GECO EF-2:4 and (**i**) mG-GECO EF-3:4 Left panels show plots of the [Ca^{2+}] dependence of the observed rate(s) (k_{obs}), right panels show stopped-flow records at the specified final [Ca^{2+}]. Fluorescence changes are normalised to F_0 of 0 and maximum of 1. For mG-GECOs with biphasic association kinetics, the slow and the fast components are represented in red and black, respectively.

Supplementary Figure S8

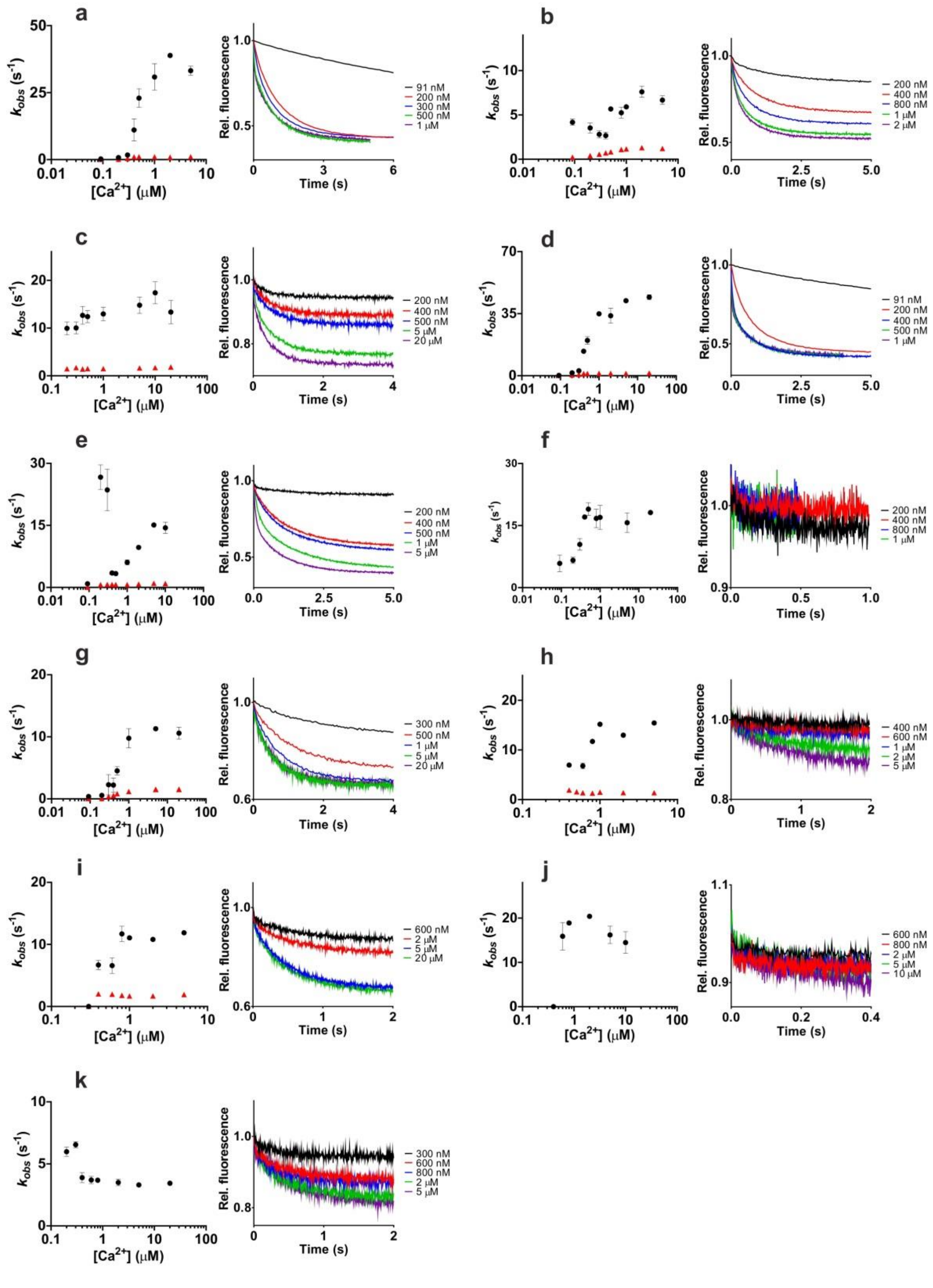

Supplementary Figure S8. Association kinetics for Ca^{2+} of (**a**) GEM-GECO, (**b**) mGEM-GECO EF-1, (**c**) mGEM-GECO EF-2, (**d**) mGEM-GECO EF-3, (**e**) mGEM-GECO EF-4, (**f**) mGEM-GECO EF-1:2, (**g**) mGEM-GECO EF-1:3, (**h**) mGEM-GECO EF-1:4, (**i**) mGEM-GECO EF-2:3, (**j**) mGEM-GECO EF-2:4, (**k**) mGEM-GECO EF-3:4. Left panels show plots of the [Ca^{2+}] dependence of the observed rate(s) (k_{obs}), right panels show stopped-flow records at the specified final [Ca^{2+}]. Fluorescence changes are normalised to F_0 of 0 and maximum of 1. For mGEM-GECOs with biphasic association kinetics, the slow and the fast components are represented in red and black, respectively.

Supplementary Figure S9

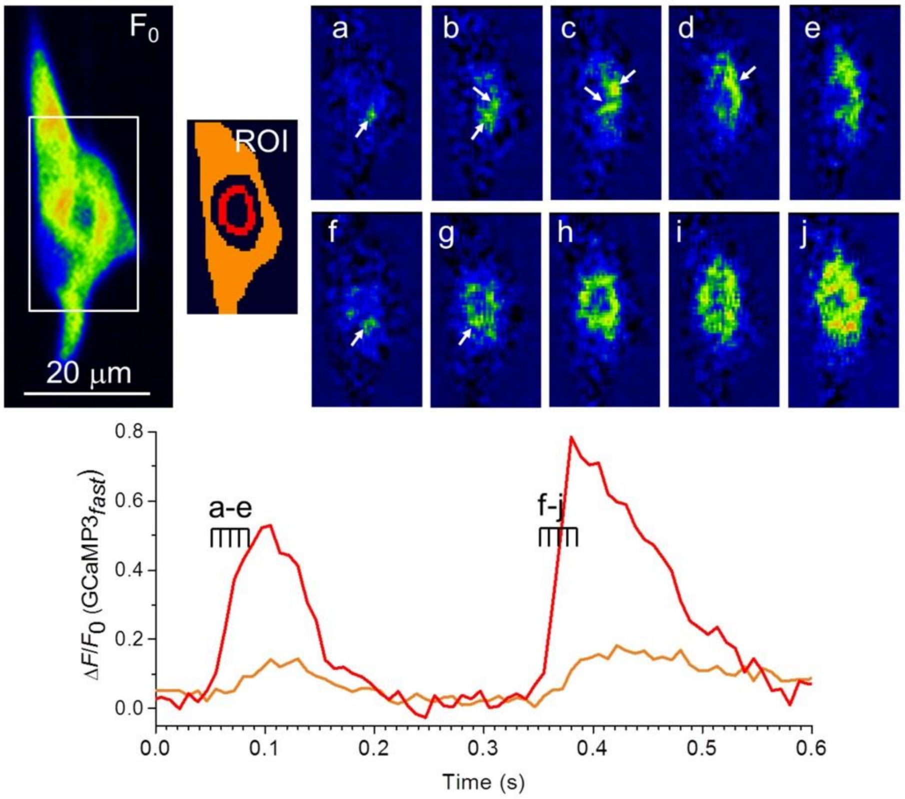

Supplementary Figure S9. Measurement of perinuclear Ca^{2+} hotspots with GCaMP3$_{fast}$ in a spontaneously pacing rat neonatal cardiomyocyte. The images show the baseline fluorescence (F_0), regions of interest (ROI), and two sequences of 5 frames (ΔF) measured at 120 Hz at the onset of two beats (**a-e** and **f-j**) at the times indicated along the traces showing intensity changes around the nucleus (red line) and in the cytosol in general (orange line). Arrows in the 1, 4, and 7 'o'clock' directions point to prominent Ca^{2+} release sites that are visible in consecutive frames and/or beats. Some local hotspots e.g. that pointed at by the 7 'o'clock' direction appear in 8.3 ms from one frame to the next and then fade away in ~20 ms after 1 or 2 additional frames. NOTE: similar time courses of local Ca^{2+} hot spots are recorded with GCaMP3$_{bright}$ too (data not shown).

ROI were chosen by first inspecting individual frames (insets a-j) and selecting localities where early release was (red) or was not (orange) apparent by ΔF. The time course of fluorescence changes in multiple points was then observed as $\Delta F/F_0$ which in the orange ROI remained low and therefore nowhere required special attention in the form of additional localized ROIs. Based on dimensions, the general morphology of neonatal cardiomyocytes, F_0-distribution, and bright field images, the red region is described as being "perinuclear". A transition region between the red and the orange ROI was omitted for clarity.

Supplementary Figure S10

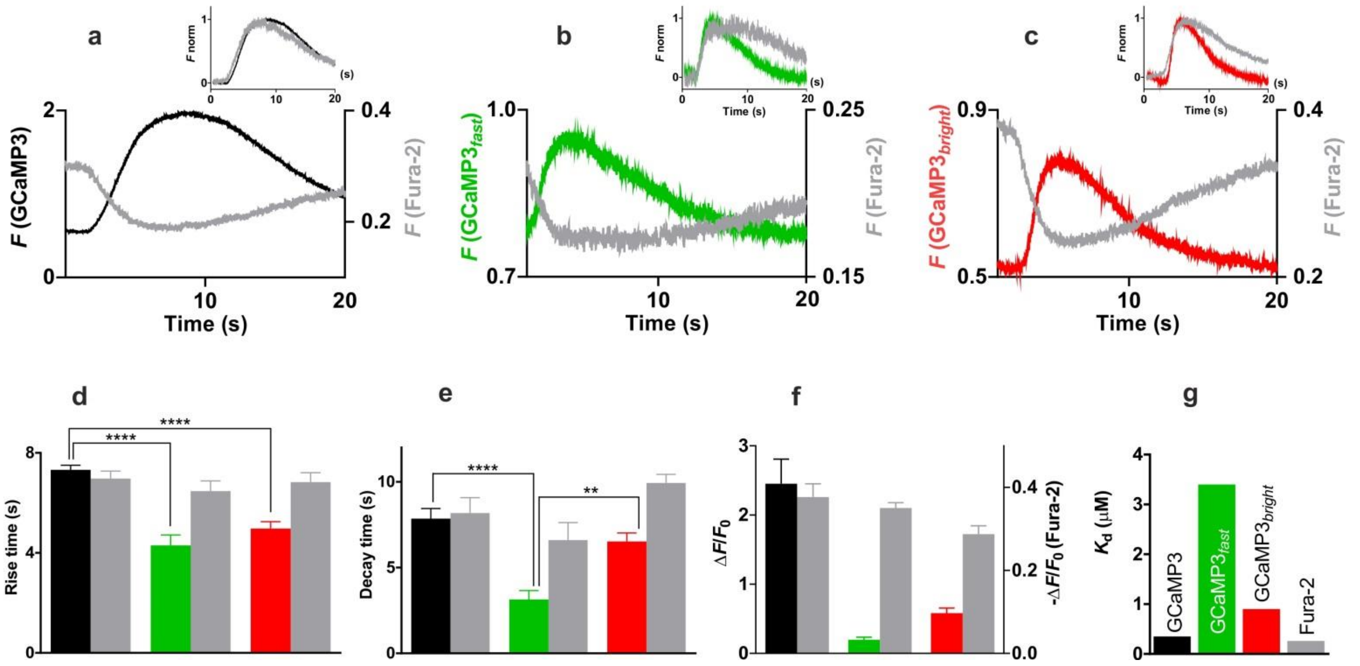

Supplementary Figure S10. Characterisation of selected mGCaMP3 probes in HEK293T cells where Ca^{2+} transients were triggered by exposure to 100 µM ATP for 5 s. (**a-c**) Representative fluorescence recordings (arbitrary units) measured with Fura-2 (▬) simultaneously with (**a**) GCaMP3 (▬), (**b**) GCaMP3*fast* (▬) or (**c**) GCaMP3*bright* (▬). Insets show comparison of normalized fluorescence responses. (**d-f**) Comparison of the average fluorescence responses of the four Ca^{2+} probes. (**d**) Fluorescence rise-times of Ca^{2+} for Fura-2 (▬) (n = 30), GCaMP3 (▬) (n = 10), GCaMP3*fast* (▬) (n = 10) and GCaMP3*bright* (▬) (n = 10) after ATP stimulation; (**e**) Fluorescence decay times (measured from peak to 30% peak amplitude) of Ca^{2+} for Fura-2 (▬) (n = 23), GCaMP3 (▬) (n = 10), GCaMP3*fast* (▬) (n = 9) and GCaMP3*bright* (▬) (n = 9) after ATP stimulation; (**f**) $\Delta F/F_0$ for the four Ca^{2+} probes, colour-coded as in (**a-c**). (**g**) *In vitro* K_d values (from **Table 1** and K_d for Fura-2 of 260 nM[30]).

Supplementary Figure S11

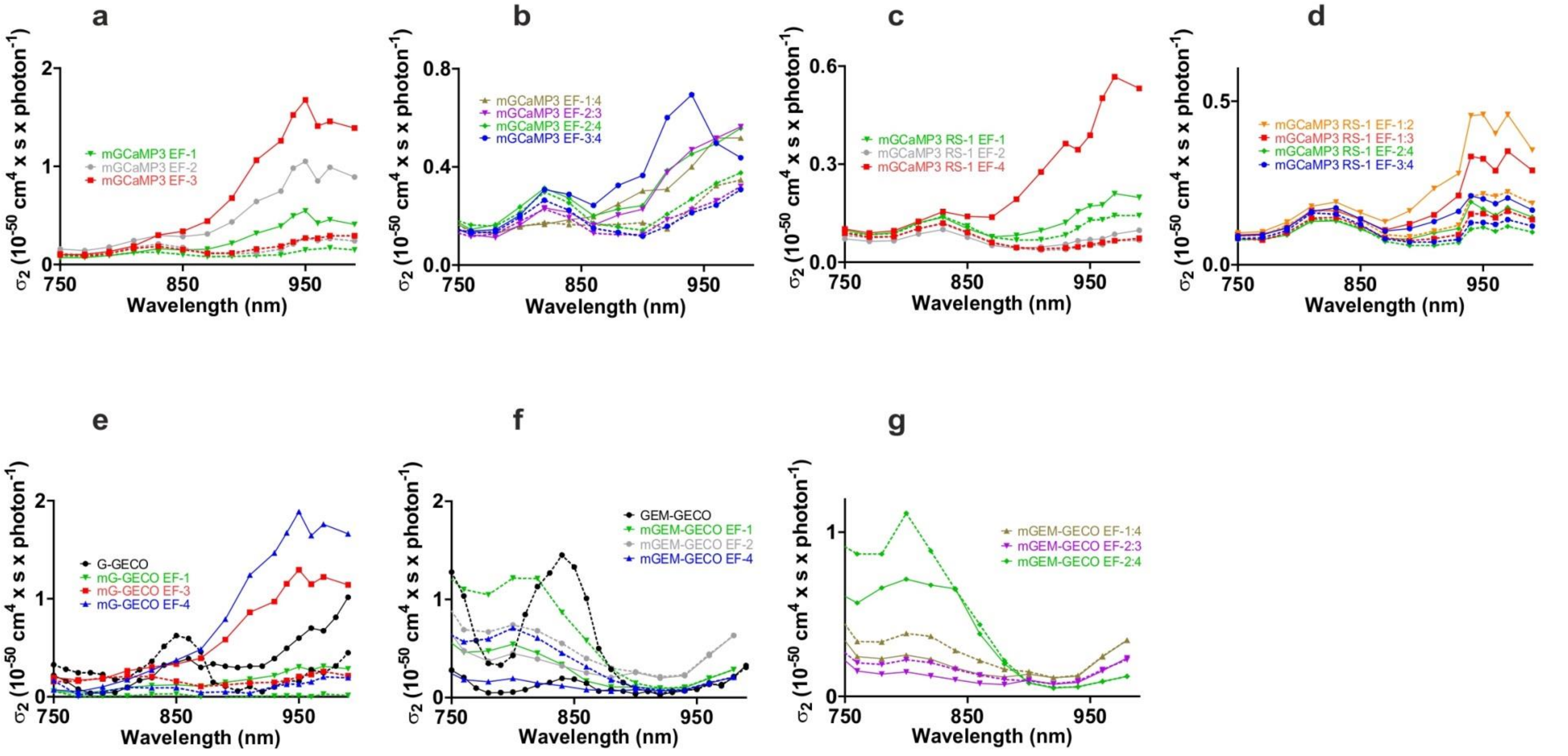

Supplementary Figure S11. Two-photon excitation spectra of mGECIs. (**a**) mGCaMP3 EF-1 (▼) and EF-2 (●) and mGCaMP3 EF-3 (■); (**b**) mGCaMP3 EF-1:4 (▲), EF-2:3 (▼), EF-2:4 (♦) and EF-3:4 (●); (**c**) mGCaMP3 RS-1 EF-1 (▼), RS-1 EF-2 (●) and RS-1 EF-4 (■); (**d**) mGCaMP3 RS-1 EF-1:2 (▼), RS-1 EF-1:3 (■), RS-1 EF-2:4 (♦) and RS-1 EF-3:4 (●); (**e**) G-GECO (●), mG-GECO EF-1 (▼), EF-3 (■) and EF-4 (▲); (**f**) mGEM-GECO (●), mGEM-GECO EF-1 (▼), EF-2 (●) and EF-4 (▲); (**g**) mGEM-GECO EF-1:4 (▲), EF-2:3(▼) and EF-2:4 (♦). Solid lines show spectra in the presence and dotted lines in the absence of Ca^{2+}.

Supplementary Figure S12

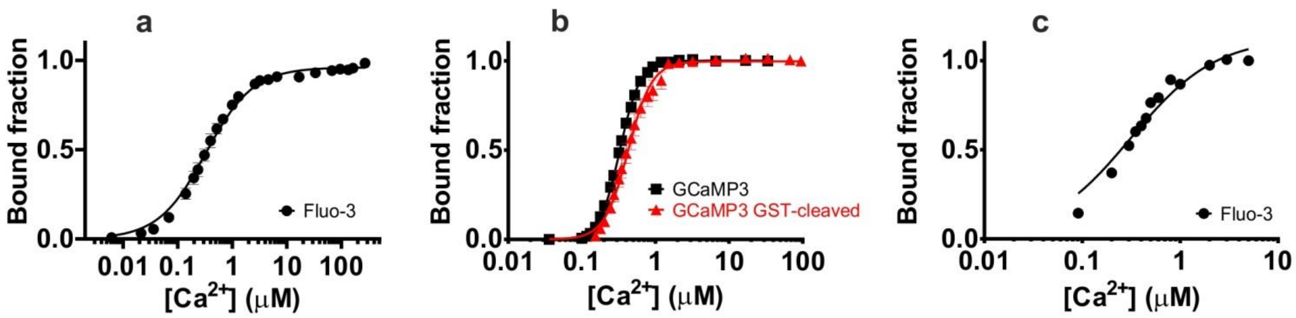

Supplementary Figure S12. Verification of [Ca^{2+}] with Fluo-3 of GST-tagged and untagged GCaMP3. (**a**) Fluo-3 titration was carried out as described in Methods. A K_d value of 329 ± 12 nM was obtained by fitting the data to a single site binding model. **(b)** Equilibrium Ca^{2+} binding of GST-tagged GCaMP3 (●) and GST-cleaved GCaMP3 (▲) were compared. K_d values of 0.42 ± 0.04 μM and 0.33 ± 0.01 μM and Hill coefficients of 2.8 and 3.2 were obtained, respectively. (**c**) Fluo-3 titration curve obtained by measuring Fluo-3 fluorescence in a series of solutions made for stopped-flow association kinetics of varied final [Ca^{2+}] as a means of verification of the calculated final [Ca^{2+}]. A K_d value of 314 ± 39 nM was obtained, not significantly different from that obtained in continuous titration experiments (see (**a**)).

Supplementary Figure S13

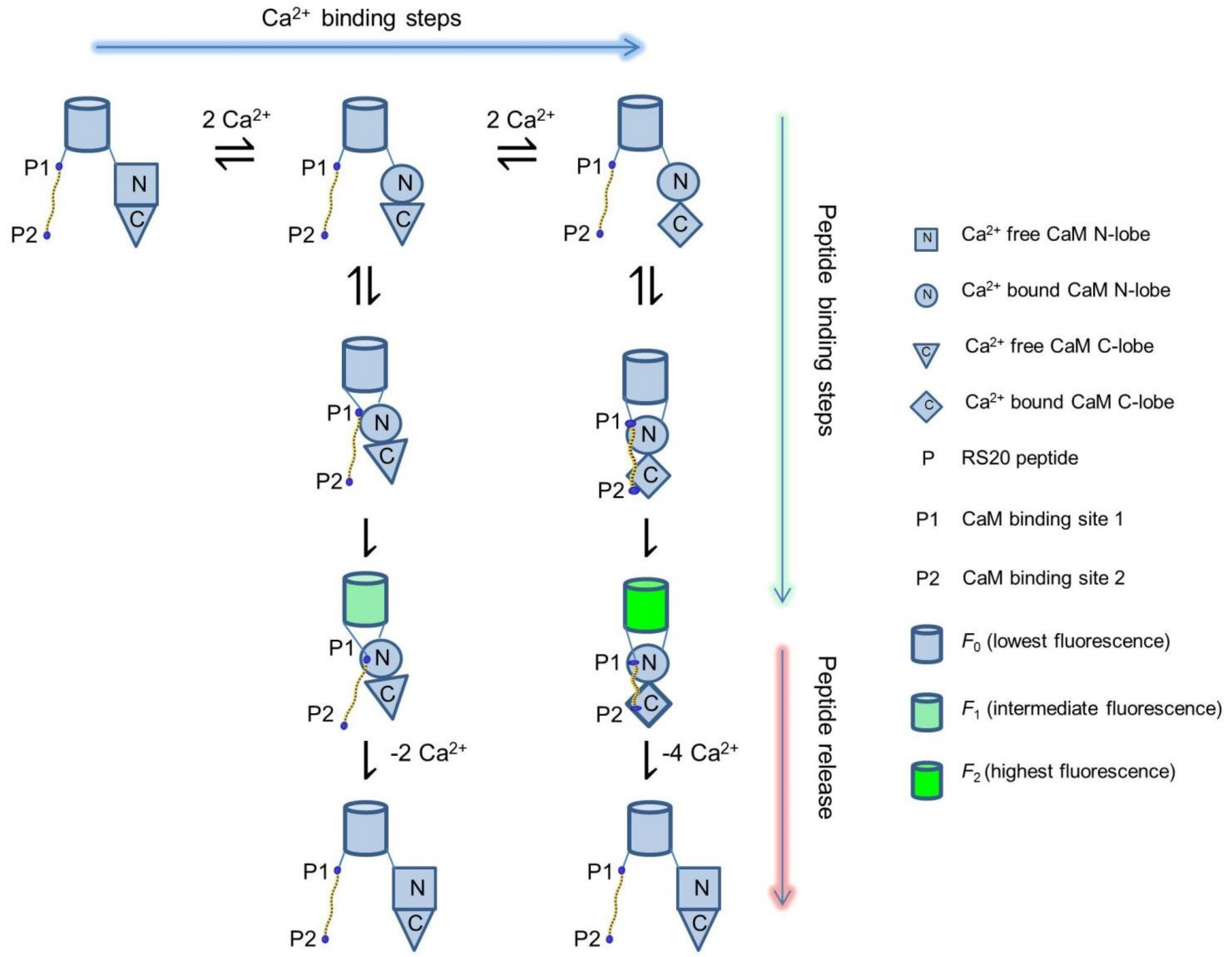

Supplementary Figure S13. Schematic model of the kinetic mechanism of GCaMP3. General model for the mechanism of Ca^{2+}-induced fluorescence of GCaMP3 depicting Ca^{2+} binding to the CaM N- and C-lobe followed by peptide binding and isomerisation leading to fluorescent states. Fluorescence is proposed to derive from the equilibrium of two states: in the first, Ca^{2+}-bound CaM N-lobe is with the P1 CaM binding site of RS20; in the second, both the N- and C-lobes of CaM are Ca^{2+}-bound binding to both the P1 and P2 sites of the peptide. Formation of the fluorescent complexes involves essentially irreversible processes. Peptide release is triggered by Ca^{2+} sequestration returning the complex to the apo-state. This general model is applied to EF-hand mutants of GCaMP3 by omitting the appropriate Ca^{2+} binding steps e.g. equation (1) (**Supplementary Fig. S14**) for the EF-1 mutant, equation (2) for EF-2 and so on.

Supplementary Figure S14

Ca^{2+} and peptide binding steps

$$Ca^{2+} + CaM(F_0) \underset{k_{-1}}{\overset{k_{+1}}{\rightleftharpoons}} Ca^{2+}.CaM \qquad (1)$$

$$Ca^{2+} + Ca^{2+}.CaM \underset{k_{-2}}{\overset{k_{+2}}{\rightleftharpoons}} Ca^{2+}{}_2.CaM \qquad (2)$$

$$Ca^{2+}{}_2.CaM + P1 \underset{k_{-3}}{\overset{k_{+3}}{\rightleftharpoons}} Ca^{2+}{}_2.CaM.P1 \qquad (3)$$

$$Ca^{2+}{}_2.CaM.P1 \underset{k_{-4}}{\overset{k_{+4}}{\rightleftharpoons}} Ca^{2+}{}_2.CaM.P1^*(F_1) \qquad (4)$$

$$Ca^{2+} + Ca^{2+}{}_2.CaM.P1 \underset{k_{-5}}{\overset{k_{+5}}{\rightleftharpoons}} Ca^{2+}{}_3.CaM.P1 \qquad (5)$$

$$Ca^{2+} + Ca^{2+}{}_3.CaM.P1 \underset{k_{-6}}{\overset{k_{+6}}{\rightleftharpoons}} Ca^{2+}{}_4.CaM.P1 \qquad (6)$$

$$Ca^{2+}{}_4.CaM.P1 + P2 \underset{k_{-7}}{\overset{k_{+7}}{\rightleftharpoons}} Ca^{2+}{}_4.CaM.P1.P2 \qquad (7)$$

$$Ca^{2+}{}_4.CaM.P1.P2 \underset{k_{-8}}{\overset{k_{+8}}{\rightleftharpoons}} Ca^{2+}{}_4.CaM.P1.P2^{**}(F_2) \qquad (8)$$

Peptide release

$$Ca^{2+}{}_4.CaM.P1.P2^*(F_2) \overset{k_{-9}}{\rightharpoonup} 4Ca^{2+} + CaM + P1P2 \qquad (9)$$

$$Ca^{2+}{}_2.CaM.P1^*(F_1) \overset{k_{-10}}{\rightharpoonup} 2Ca^{2+} + CaM + P1 \qquad (10)$$

Supplementary Figure S14. Detailed kinetic model of Ca^{2+}-induced fluorescence of GCaMP3. Equations (1) & (2) are shown as single step in **Supplementary Fig. S13**; equation (3) represents an intramolecular reaction; equation (4) describes a process in which a fluorescence increase occurs; equations (5) & (6) are shown as single step in **Supplementary Fig. S13**; equation (7) represents an intramolecular reaction; equation (8) describes a process in which a fluorescence increase occurs; Note that peptide release can occur reversibly in steps (4) & (7).

Supplementary Table S1. Summary of the biophysical characteristics and cellular fluorescence properties of selected mGECIs. Fluorescence enhancement expressed as the ratio of fluorescence intensities in the presence and absence of Ca^{2+} ($F_{(+Ca^{2+})}$ / $F_{(-Ca^{2+})}$), K_d and Hill coefficient (n) were obtained from the equilibrium calcium binding titrations at 20 °C. Maximal fluorescence rise ($k_{on(lim)}$) and decay (k_{off}) rates were obtained from the association and dissociation stopped-flow kinetics at 20 °C. The cellular fluorescence dynamic range ($\Delta F/F_0$) values were obtained from fluorescence measurements in HUVEC cells during acute exposure to ionomycin and HEK293T cells after ATP stimulation. Two-photon cross-section values in the presence and absence of Ca^{2+} were obtained from the two-photon spectra at 940 nm unless otherwise specified, using fluorescein as a reference. [a]Recorded at 920 nm. [b]Recorded at 820 nm. [c]Biphasic association kinetic records were fitted with two exponentials. The rate of each phase is given with the relative amplitudes in parentheses. [d]Measurements were made at 20, 25 and 30 °C. Rates were too fast to measure at 37 °C, thus value was extrapolated from Arrhenius plot. [e]Ratiometric ($F_{(+Ca^{2+})}$ /$F_{(-Ca^{2+})})_{exc\ 492}$ /($F_{(+Ca^{2+})}$ /$F_{(-Ca^{2+})})_{exc\ 392}$. [f]Recorded at 930 nm. n.d. stands for not determined.

Protein	$F_{(+Ca^{2+})}$ / $F_{(-Ca^{2+})}$	K_d (μM)	n	$k_{on(lim)}$[c] (s^{-1}) 20 °C	$k_{on(lim)}$[c] (s^{-1}) 37 °C	k_{off}[c] (s^{-1}) 20 °C	k_{off}[c] (s^{-1}) 37 °C	σ_2 (10^{-50} cm^4 x s x $photon^{-1}$) - Ca^{2+}	σ_2 (10^{-50} cm^4 x s x $photon^{-1}$) + Ca^{2+}	$\Delta F/F_0$ (HUVEC)	$\Delta F/F_0$ (HEK293T)
**GCaMP3**	6.3	0.33 ± 0.01	3.2 ± 0.1	20 ± 1	19 ± 1	1.5 ± 0.1	5.3 ± 1	0.06[f]	0.76[f]	5.6	2.5 ± 0.4
**GCaMP3*fast***	8.8	2.8 ± 0.3	4.3 ± 0.2	145 ± 2	743[d]	62 ± 2	210 ± 6	0.10	0.47	3.4	0.2 ± 0.04
**GCaMP3*bright***	14	0.93 ± 0.01	4.7 ± 0.1	135 ± 8 (0.5)<br>19 ± 1 (0.5)	139 ± 41 (0.5)<br>26 ± 2 (0.5)	2.8 ± 0.1	15 ± 0.2	0.22	2.00	5.6	0.6 ± 0.1
**mGCaMP3 EF-3**	7.2 ± 0.2	0.50 ± 0.01	4.2 ± 0.1	148 ± 14 (0.5)<br>24 ± 1 (0.5)	155 ± 55 (0.5)<br>27 ± 3 (0.5)	3.1 ± 0.1	12 ± 1	0.23	1.52	6.7	1.3 ± 0.1
**mGCaMP3 EF-3:4**	2.6 ± 0.2	2.4 ± 0.1	2.8 ± 0.2	141 ± 6	235[d]	99 ± 1	283 ± 10	0.21	0.69	3.9	0.1 ± 0.03
**mGCaMP3 RS-1**	10.4 ± 0.2	0.95 ± 0.01	3.4 ± 0.1	67 ± 1	105 ± 2	2.5	14.4 ± 0.4	n.d.	n.d.	n.d.	n.d.
**mGCaMP3 RS-1 EF-4**	11.1 ± 0.3	5.6 ± 0.1	3.8 ± 0.1	86 ± 2 (0.6)<br>30 ± 2 (0.4)	189 ± 10	66 ± 1	302 ± 3	0.08	0.55	3.9	0.4 ± 0.1
**G-GECO**	12.7 ± 0.1	0.63 ± 0.01	3.3 ± 0.1	8.3 ± 0.1	n.d.	1.8 ± 0.1	n.d.	0.16	0.50	11.2	n.d.
**mG-GECO EF-3**	12.4 ± 0.4	1.3 ± 0.1	2.7 ± 0.1	44 ± 1 (0.5)<br>6.7 ± 0.1 (0.5)	n.d.	1.7 ± 0.1	n.d.	0.17	1.15	6.7	n.d.
**mG-GECO EF-4**	14.2 ± 1.0	1.7 ± 0.1	2.7 ± 0.2	26 ± 2 (0.3)<br>6.0 ± 0.2 (0.7)	n.d.	5.4 ± 0.1	n.d.	0.13	1.67	4.5	n.d.
**GEM-GECO**	107 ± 2[e]	0.13 ± 0.01	3.6 ± 0.1	33 ± 3 (0.3)<br>1.0 ± 0.1 (0.7)	n.d.	0.08 ± 0.01	n.d.	1.13[a]	0.13[a]	n.d.	n.d.
**mGEM-GECO EF-1**	96 ± 5[e]	0.65 ± 0.01	2.2 ± 0.1	7.1 ± 0.4 (0.5)<br>1.3 ± 0.1 (0.5)	n.d.	0.30 ± 0.01	n.d.	1.21[b]	0.45[b]	n.d.	n.d.
**mGEM-GECO EF-2**	41 ±3[e]	1.8 ± 0.1	2.0 ± 0.1	15 ± 1 (0.3)<br>0.9 ± 0.1 (0.7)	n.d.	3.3 ± 0.1	n.d.	0.68[b]	0.39[b]	n.d.	n.d.

Supplementary Table S2. Summary of the biophysical characteristics and cellular fluorescence properties of other mGECIs. Fluorescence enhancement expressed as the ratio of fluorescence intensities in the presence and absence of Ca^{2+} ($F_{(+Ca^{2+})}$ / $F_{(-Ca^{2+})}$), K_d and Hill coefficient (n) were obtained from the equilibrium calcium binding titrations at 20 °C. Maximal fluorescence rise ($k_{on(lim)}$) and decay (k_{off}) rates were obtained from the association and dissociation stopped-flow kinetics at 20 °C. The cellular fluorescence dynamic range ($\Delta F/F_0$) values were obtained from fluorescence measurements in HUVEC cells during acute exposure to ionomycin and HEK293T cells after ATP stimulation. Two-photon cross-section values in the presence and absence of Ca^{2+} were obtained from the two-photon spectra at 940 nm unless otherwise specified, using fluorescein as a reference. [a]Recorded at 950 nm. [b]Recorded at 920 nm. [c]Recorded at 820 nm. [d]Biphasic assiciation kinetic records were fitted with two exponentials. The rate of each phase is given with the relative amplitudes in parentheses. Error represents the standard error of the estimate determined from the average of three records. [e]Ratiometric (F(+Ca2+) /$F(_{-Ca^{2+}}))_{exc\ 492}$ /$(F(_{+Ca^{2+}})$ /$F(_{-Ca^{2+}}))_{exc\ 392}$. [f]Recorded at 930 nm. n.d. stands for not determined and n.d.c. for no detectable change.

Protein	$F_{(+Ca^{2+})}$ / $F_{(-Ca^{2+})}$	K_d (μM)	*n*	$k_{on(lim)}$[d] (s^{-1})	k_{off}[d] (s^{-1})	σ_2 (10^{-50} cm^4 x s x $photon^{-1}$) - Ca^{2+}	+ Ca^{2+}	$\Delta F/F_0$ (HUVEC)
**GCaMP3**	6.3	0.33 ± 0.01	3.2 ± 0.1	20 ± 1	1.5 ± 0.1	0.06[f]	0.76[f]	5.6
**GCaMP3*fast***	8.8	2.8 ± 0.3	4.3 ± 0.2	145 ± 2	62 ± 2	0.10	0.47	3.4
**GCaMP3*bright***	14	0.93 ± 0.01	4.7 ± 0.1	135 ± 8 (0.5)<br>19 ± 1 (0.5)	2.8 ± 0.1	0.22	2.00	5.6
**mGCaMP3 EF-1**	2.7 ± 0.1	1.1 ± 0.1	4.2 ± 0.2	29 ± 1	14 ± 1	0.30[a]	1.093[a]	3.2
**mGCaMP3 EF-2**	2.5 ± 0.1	1.4 ± 0.1	3.6 ± 0.2	9 ± 0.3	15 ± 1	0.21	0.98	3.7
**mGCaMP3 EF-1:2**	1.3 ± 0.1	n.d.c.	n.d.c.	n.d.c.	n.d.c.	1.04	1.05	n.d.
**mGCaMP3 EF-1:3**	1.5 ± 0.1	4.8 ± 0.3	2.3 ± 0.2	n.d.c.	27 ± 1	0.24	0.36	3.2
**mGCaMP3 EF-1:4**	3.1 ± 0.2	2.0 ± 0.1	2.9 ± 0.2	21 ± 1	33 ± 1	0.23	0.40	3.7
**mGCaMP3 EF-2:3**	1.7 ± 0.1	2.0 ± 0.1	3.3 ± 0.2	12 ± 0.3	18 ± 1	0.22	0.47	3.2
**mGCaMP3 EF-2:4**	3.5 ± 0.1	1.5 ± 0.1	2.9 ± 0.1	9 ± 0.2	31 ± 1	0.27	0.45	3.2
**mGCaMP3 RS-1 EF-1**	2.6 ± 0.1	1.3 ± 0.1	1.3 ± 0.1	n.d.c.	63 ± 6 (0.4)<br>4.3 ± 0.4 (0.6)	0.15	0.23	n.d.
**mGCaMP3 RS-1 EF-2**	1.9 ± 0.1	1.2 ± 0.1	1.1 ± 0.1	n.d.	50 ± 4 (0.5)<br>4.3 ± 0.7 (0.5)	0.06	0.09	2.7
**mGCaMP3 RS-1 EF-1:2**	2.1 ± 0.1	0.94 ± 0.02	1.7 ± 0.1	n.d.c.	41 ± 4 (0.2)<br>2.6 ± 0.1 (0.8)	0.30	0.64	n.d.
**mGCaMP3 RS-1 EF-1:3**	1.8 ± 0.1	2.3 ± 0.1	1.6 ± 0.1	n.d.c.	333 ± 83	0.24	0.52	n.d.
**mGCaMP3 RS-1 EF-1:4**	2.5 ± 0.2	3.4 ± 0.1	2.2 ± 0.1	n.d.c.	414 ± 62	0.13	0.16	3.2
**mGCaMP3 RS-1 EF-2:3**	1.7 ± 0.1	2.6 ± 0.1	1.7 ± 0.1	n.d.c.	204 ± 26	0.17	0.24	n.d.
**mGCaMP3 RS-1 EF-2:4**	2.0 ± 0.1	5.3 ± 0.1	1.5 ± 0.1	n.d.c.	208 ± 29	0.17	0.31	n.d.
**mGCaMP3 RS-1 EF-3:4**	3.1 ± 0.1	3.3 ± 0.1	3.3 ± 0.1	n.d.c.	283 ± 10	0.26	0.42	2.8
**mG-GECO EF-1**	2.3 ± 0.1<br>2.7 ± 0.1	1.7 ± 0.1<br>294 ± 38	3.0 ± 0.2<br>0.8 ± 0.1	18 ± 0.5	18 ± 1 (0.7)<br>2.4 ± 0.1 (0.3)	0.01	0.26	4.8
**mG-GECO EF-2**	1.1 ± 0.1<br>2.1 ± 0.1	2.3 ± 0.2<br>534 ± 15	3.6 ± 1<br>1.1 ± 0.1	5 ± 0.2	12 ± 1 (0.8)<br>1.5 ± 0.1 (0.2)	0.23	0.31	5.3
**mG-GECO EF-1:2**	n.d.c.	n.d.c.	n.d.c.	n.d.c.	2.6 ± 0.4 (0.8)<br>0.9 ± 0.2 (0.2)	0.08	0.08	n.d.
**mG-GECO EF-1:3**	1.4 ± 0.1<br>2 ± 0.1	4.0 ± 0.3<br>873 ± 91	2.5 ± 0.3<br>1.0 ± 0.1	n.d.c.	10 ± 1	1.29[c]	1.30[c]	n.d.
**mG-GECO EF-1:4**	1.9 ± 0.1<br>3.9 ± 0.1	3.0 ± 0.2<br>842 ± 128	2.6 ± 0.3<br>1.2 ± 0.1	16 ± 1	24 ± 1	0.84	0.84	4.2
**mG-GECO EF-2:3**	2.2 ± 0.1	1770 ± 211	1.0 ± 0.1	5 ± 0.3	13 ± 1 (0.7)<br>1.2 ± 0.1 (0.3)	0.95	1.00	6.4
**mG-GECO EF-2:4**	1.4 ± 0.1<br>3.4 ± 0.2	4.1 ± 0.6<br>1084 ± 164	1.6 ± 0.3<br>1.0 ± 0.1	5 ± 0.3	21 ± 2 (0.8)<br>3.2 ± 2.5 (0.2)	0.91[b]	0.96[b]	6.9
**mG-GECO EF-3:4**	3.2 ± 0.2<br>6.2 ± 0.4	1.1 ± 0.1<br>1033 ± 50	4.3 ± 0.2<br>1.4 ± 0.1	43 ± 2	74 ± 3	1.58	1.65	5.6
**mGEM-GECO EF-3**	108 ± 8[e]	1.9 ± 0.1	3.1 ± 0.1	39 ± 3 (0.5)<br>1 ± 0.1 (0.5)	0.08 ± 0.01	0.12[b]	0.12[b]	n.d.
**mGEM-GECO EF-4**	92 ± 13[e]	0.92 ± 0.01	2.7 ± 0.1	15 ± 1 (0.5)<br>1 ± 0.1 (0.5)	1.04 ± 0.01	0.61[b]	0.15[b]	n.d.
**mGEM-GECO EF-1:2**	n.d.c.	n.d.c.	n.d.c.	17 ± 1	2.6 ± 0.1	0.81[c]	0.73[c]	n.d.
**mGEM-GECO EF-1:3**	80 ± 2[e]	0.91 ± 0.01	3.3 ± 0.1	11 ± 1 (0.2)<br>1 ± 0.1 (0.8)	0.30 ± 0.01	0.13[c]	0.13[c]	n.d.
**mGEM-GECO EF-1:4**	33 ± 2[e]	2.7 ± 0.1	2.6 ± 0.1	15 ± 1 (0.5)<br>1 ± 0.1 (0.5)	2.8 ± 0.1	0.37[c]	0.23[c]	n.d.
**mGEM-GECO EF-2:3**	42 ± 2[e]	2.2 ± 0.1	2.4 ± 0.1	11 ± 0.3 (0.4)<br>2 ± 0.1 (0.6)	4.4 ± 0.1	0.21[b]	0.13[b]	n.d.
**mGEM-GECO EF-2:4**	31 ± 2[e]	232 ± 16	1.0 ± 0.1	17 ± 1	8.3 ± 0.2	0.89[c]	0.68[c]	n.d.
**mGEM-GECO EF-3:4**	20 ± 3[e]	n.d.c.	n.d.c.	4 ± 0.1	2.3 ± 0.1	0.35[c]	0.30[c]	n.d.

Supplementary Table S3. A set of best-fit parameters for the kinetic model of GCaMP3 and a selection of mGCaMP3.

[a]mGCaMP3 EF-3:4 best fit required that the values for k_{-1} and k_{-2} to be orders of magnitude smaller than for GCaMP3 and its EF-hand mutants. Similar behaviour characterised the RS-1 peptide mutants for k_{-1}. [b]Irreversible steps are indicated by the small values of rate constants best fit for reversal of peptide binding or isomerisation steps.

Protein	k_{+1} (μM^{-1} s^{-1})	k_{-1} (s^{-1})	k_{+2} (μM^{-1} s^{-1})	k_{-2} (s^{-1})	k_{+3} (μM^{-1} s^{-1})	k_{-3} (s^{-1})	k_{+4} (s^{-1})	k_{-4} (s^{-1})	k_{+5} (μM^{-1} s^{-1})	k_{-5} (s^{-1})	k_{+6} (μM^{-1} s^{-1})	k_{-6} (s^{-1})	k_{+7} (μM^{-1} s^{-1})	k_{-7} (s^{-1})	k_{+8} (s^{-1})	k_{-8} (s^{-1})	F_1	F_2	K_d (μM)
**GCaMP3**	770	1.10E+03	1.50E+04	1.12E+05	1.01E+03	4.90E-05	1.49E+03	168	84	33	25	5.00E-05	1.19E+03	47.5	58	70	21	31	0.351
**mGCaMP3 EF-3**	770	4.16E+03	1.50E+04	1.82E+03	1.01E+03	2.37E-05	146	199	-	-	84	2.00E-04	1.19E+03	2.70E-03	300	10	28	13	0.511
**GCaMP3*bright***	770	4.16E+03	1.50E+04	2.60E+03	1.01E+03	2.37E-05	146	86	84	1.52E-06	-	-	1.19E+03	0.05	300	1.27	18	17	0.916
**mGCaMP3 EF-3:4**	700	0.15	1.21E+03	0.4	268	4.4	378	548	-	-	-	-	-	-	-	-	5	-	2.33
**mGCaMP3 RS-1**	770	13.8[a]	1.50E+04	1.46E+04	504	38	1.25E+03	21	118	5	25	247	1.86E+03	9.60E-02	1.25E+03	8.0E-03[b]	5.6	5	0.92
**GCaMP3*fast***	770	2.9	1.50E+04	3.83E+04	786	97	65	176	-	-	118	31	960	2.50E-07	368	1.81	27	4.5	3.36
**mGCaMP3 RS-1 EF-4**	770	2.9	1.50E+04	3.83E+04	786	323	16	74	118	23.6	-	-	840	2.90E-08	136	8	53	6.4	5.55

Supplementary Table S4. Absorption properties of mGECI. Extinction coefficients at 280 nm (ε_{280}) were calculated from the amino acid sequence and the extinction coefficient at 400 nm (ε_{400}) was obtained from the A_{280}/A_{400} ratio.

Protein	10^{-4} x $\varepsilon_{0\ (280\ nm)}$	10^{-4} x $\varepsilon_{0\ (400\ nm)}$
GCaMP3		2.21
mGCaMP3 EF-1		3.13
mGCaMP3 EF-2		3.41
mGCaMP3 EF-3		3.75
GCaMP3$_{bright}$		3.00
mGCaMP3 EF-1:2	7.51	3.12
mGCaMP3 EF-1:3		3.48
mGCaMP3 EF-1:4		3.75
mGCaMP3 EF-2:3		2.61
mGCaMP3 EF-2:4		3.95
mGCaMP3 EF-3:4		3.00
mGCaMP3 RS-1		2.13
mGCaMP3 RS-1 EF-1		2.62
mGCaMP3 RS-1 EF-2		2.40
GCaMP3$_{fast}$		2.63
mGCaMP3 RS-1 EF-4		2.67
mGCaMP3 RS-1 EF-1:2	7.07	2.67
mGCaMP3 RS-1 EF-1:3		2.65
mGCaMP3 RS-1 EF-1:4		2.45
mGCaMP3 RS-1 EF-2:3		2.92
mGCaMP3 RS-1 EF-2:4		2.85
mGCaMP3 RS-1 EF-3:4		2.97
G-GECO		2.76
mG-GECO EF-1		1.99
mG-GECO EF-2		1.50
mG-GECO EF-3		2.07
mG-GECO EF-4		3.21
mG-GECO EF-1:2	7.66	2.09
mG-GECO EF-1:3		1.43
mG-GECO EF-1:4		1.86
mG-GECO EF-2:3		3.57
mG-GECO EF-2:4		2.33
mG-GECO EF-3:4		1.16
GEM-GECO		3.14
mGEM-GECO EF-1		3.22
mGEM-GECO EF-2		3.94
mGEM-GECO EF-3		3.28
mGEM-GECO EF-4		2.50
mGEM-GECO EF-1:2	7.62	1.83
mGEM-GECO EF-1:3		1.35
mGEM-GECO EF-1:4		1.83
mGEM-GECO EF-2:3		2.10
mGEM-GECO EF-2:4		2.08
mGEM-GECO EF-3:4		2.90
